# Gender identity and cannabis use in Canada and the United States: cross-sectional analysis of the International Cannabis Policy Study

**DOI:** 10.1186/s42238-026-00452-6

**Published:** 2026-06-12

**Authors:** Anastasia Marquette, Lorraine Greaves, Maryam Iraniparast, Samantha Rundle, Ava Kucera, David Hammond

**Affiliations:** 1 School of Public Health Sciences, University of Waterloo, 200 University Avenue West, Waterloo, ON N2L 3G1 Canada; 2 Centre of Excellence for Women’s Health, E209-4500 Oak Street, Vancouver, BC Canada; 3 School of Population and Public Health, Faculty of Medicine, University of British Columbia, Vancouver, BC Canada

**Keywords:** Gender, Cannabis, Addiction, Norms

## Abstract

**Background:**

Gender identity influences patterns of substance use; however, few population-level studies have included transgender and gender-diverse identities when examining patterns of cannabis use by gender identity.

**Methods:**

Data are from the International Cannabis Policy Study’s national, repeat cross-sectional surveys conducted annually between 2018–2023 in Canada and the United States. Weighted logistic and linear regression models examined differences by gender identity among 281 533 respondents aged 16–65 (including 2 445 transgender respondents and 783 with gender-diverse identities) for three primary outcomes: patterns of cannabis use, personal effects from cannabis, and comfort consuming cannabis across social settings.

**Results:**

Daily consumption was reported by approximately 14% of cisgender men, transgender men, and people with gender-diverse identities, with less cisgender women (12%, OR = 0.74, CI95 = 0.72,0.77; *p* < .001) and transgender women (12%, OR = 0.76, CI95 = 0.59,1.00; *p* = .047) reporting daily consumption. Approximately 12% of cisgender men, transgender men, and transgender women reported being ‘very addicted’ to cannabis. Transgender women (6%, OR = 0.57, CI95 = 0.53,0.62; *p* < .001) and people with gender-diverse identities (6%, OR = 0.47, CI95 = 0.24,0.91; *p* = .025) were less likely to report being ‘very addicted’ compared to cisgender men. People with gender-diverse identities reported greater positive personal effects from cannabis compared to cisgender men (*β*=0.53, CI95 = 0.21,0.86, *p* = .001), with no differences between cisgender men, cisgender women, transgender men, and transgender women. Comfort consuming cannabis across social settings was lower among cisgender women than cisgender men (*β*=-0.21, CI95 = -0.23,-0.19; *p* < .001), with no differences between cisgender men, transgender men, and transgender women.

**Conclusions:**

Frequent and problematic patterns of cannabis use were more common among cisgender men, transgender men, and transgender women. These disparities warrant further consideration in both research and policy settings.

**Supplementary Information:**

The online version contains supplementary material available at 10.1186/s42238-026-00452-6.

## Background

Gender identity refers to one’s socio-cultural identity on a spectrum of identities, including woman, man, non-binary, and gender-diverse (Kaufman et al. 2023). Gender identity is a social construct, distinct from sex, a biological construct reflected in characteristics such as hormones, genes, physiology, and anatomy. Sex is typically assigned at birth based on observation of one’s sex characteristics (i.e., female, male, intersex) (Kaufman et al. 2023). The term ‘cisgender’ refers to identities where gender identity is assumed to be aligned with one’s sex assigned at birth, whereas ‘transgender’ typically refers to gender identities incongruent with one’s sex assigned at birth (Lachance et al 2017). ‘Gender-diverse’ is used to describe people with gender identities outside of the binary of ‘woman’ and ‘man’, including people with ‘non-binary’, ‘gender-queer’, and ‘Two-Spirit’ identities, among others (Lachance et al 2017). Gender identity is just one aspect of the social construct of gender, which includes roles, relations, and institutional gender in addition to identity, all of which affect cannabis use (Hemsing and Greaves 2020). Gender identity reflects perceptions of self as men, women or gender-diverse, and one’s incorporation and expression of hegemonic femininities and masculinities.

A vast body of literature has demonstrated the importance of both sex and gender for a wide variety of health outcomes, particularly with respect to differences between men and women, identifying sex and gender related factors and mechanisms underlying diseases and conditions, sex/gender interactions, intersectional issues, and sexual and gender minority health (Kaufman et al. 2023; Greaves and Ritz 2022). For instance, men and women experience different roles and responsibilities resulting from social norms and expectations, different levels of societal power, and access to opportunities and resources, such as healthcare treatment, income or access to substances (Kaufman et al. 2023; Fonseca et al. 2021).

Both sex and gender have wide-ranging and important implications for understanding substance use (Greaves et al 2021). Generally, men are more likely to use substances such as alcohol, cannabis, and opioids; however differences between men and women have recently narrowed for cannabis consumption, tobacco smoking, and vaping (Fonseca et al. 2021; Slade et al. 2016; McHugh et al. 2017). Most literature to date on gender differences has relied upon identifying or asking about sex, although a growing number of studies have begun to ask about and examine gender identity in addition to sex.

Current evidence indicates that substance use prevalence among transgender and gender-diverse groups varies widely depending upon the substance and population; however, most studies have indicated higher rates of substance use in these groups (Fahey et al. 2023; Connolly and Gilchrist 2020; Ruppert et al. 2021; Somé et al. 2022; Sunder et al. 2026). For example, reports of the prevalence of problematic alcohol consumption among transgender and gender-diverse groups range from 11–59%; an estimated 1.5–3 times greater than their cisgender counterparts (Fahey et al. 2023; Connolly and Gilchrist 2020; Ruppert et al. 2021).

Gendered influences on cannabis use are well-established (Centre of Excellence for Women’s Health 2021). In addition to higher prevalence of use, men who consume cannabis are more likely to report daily use and consume greater quantities compared to women (Hemsing and Greaves 2020; Greaves and Hemsing 2020; Matheson and Le Foll 2023). The limited research on transgender and gender-diverse groups indicate greater levels of cannabis use compared to their cisgender counterparts (Hemsing and Greaves 2020; Ruppert et al. 2021; Somé et al. 2022; Sunder et al. 2026; Government of Canada 2024). For example, a scoping review identified multiple estimates of past 30-day cannabis consumption among transgender and gender-diverse populations, which ranged around 30% (Ruppert et al. 2021). This is more than twice the estimates of past 30-day cannabis consumption among cisgender populations in the same studies, which were approximately 13% (Ruppert et al. 2021).

The extent to which the impact of cannabis policies may differ by gender identity is an important question, particularly given the increasing number of jurisdictions that have legalized medical and ‘recreational’ cannabis (Government of Canada 2022; Substance Abuse and Mental Health Services Administration 2023). The use, growth, and purchase of recreational cannabis is legal in a growing number of countries, including Uruguay (2014), Canada (2018), and Germany (2024), as well as 24 states within the United States (Government of Canada 2018; CDC 2024). A 2016 cross-sectional survey conducted after recreational legalization in Washington state reported that males were more likely to report consuming cannabis for recreational reasons, whereas females were more likely to report consuming cannabis for medical purposes (Cuttler et al. 2016). To our knowledge, no population-level study has examined the use of cannabis for recreational versus medical purposes among individuals with transgender and gender-diverse identities.

Rates of problematic cannabis use also differ by sex category and/or gender identity. Cannabis use disorder (CUD) is a problematic outcome of cannabis use; with individuals experiencing issues with cravings, withdrawal, impairments to work and personal life, and an inability to cease consumption, as per the Diagnostic and Statistical Manual of Mental Disorders, Fifth-Edition’s criteria (American Psychiatric Association 2013). People with CUD also report lower quality-of-life, mental, and physical health (Greaves and Hemsing 2020). One systematic review and meta-analysis examined 10 studies focused on the relationship between CUD and sex, and concluded that the likelihood of CUD was higher among males versus females (Leung et al. 2020). In addition to echoing these findings, another scoping review also found that the interval between the onset of cannabis use and the onset of CUD is shorter among females (Greaves and Hemsing 2020). To our knowledge, few population-level studies have estimated the prevalence of CUD among transgender and gender-diverse groups. However, one review has identified some evidence that people identifying as transgender or gender-diverse are at the greatest risk of developing CUD compared to sexual minority women (Dyar 2022). It has been suggested that higher rates of problematic cannabis use among gender minorities may be a product of these individuals using cannabis to cope with gender-related discrimination (Hemsing and Greaves 2020; Dyar et al. 2019; Flentje et al. 2024; Puckett et al 2025). A 2024 longitudinal study found that gender-diverse and transgender individuals who experienced discrimination and/or victimization in the past year had more problems related to their cannabis use (measured via the NIDA-Modified Alcohol, Smoking and Substance Involvement Screening Test scores), thereby indicating greater risk for CUD (Flentje et al. 2024). A similar pattern was observed among people with higher levels of internalized stigma related to their own sexual and gender minority status: those with greater internalized stigma were at greater risk for CUD (Flentje et al. 2024).

Gender, including gender identity can also influence social norms around substance use. Social norms influence decisions to use cannabis, frequency of use, types of products used, and consumption levels (Greaves and Hemsing 2020). For example, women tend to consume cannabis products with lower tetrahydrocannabinol (THC) and prefer cannabis edibles to a greater extent than men, likely because they are more discrete (Greaves and Hemsing 2020). Additionally, a 2023 study using cross-sectional survey data found that females reported being less comfortable openly consuming cannabis compared to males (Winfield-Ward and Hammond 2024). Literature reviews identify that cannabis can facilitate the expression of gender identity. Cannabis use is typically perceived as more masculine, whereas abstaining, or using less, is perceived as more feminine (Hemsing and Greaves 2020). Multiple longitudinal studies have found that adolescents who act according to dominant masculine social norms report greater cannabis use over time (Hemsing and Greaves 2020; Matheson and Le Foll 2023). To our knowledge, only one qualitative study consisting of structured interviews has specifically assessed this relationship among transgender and gender-diverse populations. Consistent with quantitative studies, the study concluded that cannabis is used to express and understand one’s gender identity, including expressions of masculinity (Barborini et al. 2024).

Overall, most population-level cannabis studies lack the necessary questions to examine gender identity, while studies that do adequately gather data on both sex and gender identity typically lack sufficient gender-diverse sample sizes to support quantitative analyses. Estimates of cannabis use patterns are therefore limited or non-existent among transgender and gender-diverse groups. Social norms among these populations are also understudied, as are the relevant sex-related factors affecting use. The incorrect use of sex interchangeably with gender, and specifically gender identity, is common in the literature and masks the impacts of sex, gender, and gender identity in producing cannabis-related behaviours. This limits our knowledge of the relationship between gender identity and cannabis. The current study sought to examine the relationship between gender identity and patterns of cannabis use in Canada and the US, with three main objectives: 1) to examine patterns of cannabis use by gender identity; 2) to examine the personal effects of cannabis use by gender identity; and 3) to examine differences in social norms related to cannabis use by gender identity.

## Methods

### Study design

Data are from the International Cannabis Policy Study’s (ICPS) national repeat cross-sectional surveys conducted annually in Canada and the US (Hammond 2018, 2019, 2020, 2021, 2022, 2023). The current analysis includes data collected from participants aged 16–65 years via self-completed web-based surveys conducted in August-October 2018, September–October 2019, September–November 2020, September–November 2021, September–October 2022, and September–October 2023. A non-probability sample of respondents was recruited through the Nielsen Consumer Insights Global Panel and their partners’ panels. The Nielsen Panels are recruited using a variety of probability and non-probability sampling methods. For the ICPS surveys, Nielsen draws stratified random samples from online panels, with quotas based on age and province/state of residence. All participants younger than 18 years were recruited through their parents, who provided consent as did the respondent. Upon completion, respondents received remuneration in accordance with their panel’s usual incentive structure. Monetary incentives have been shown to increase response rates and decrease response bias in subgroups under-represented in surveys, including disadvantaged subgroups (Groves et al. 2009). The cooperation rate, which was calculated based on American Association for Public Opinion Research Cooperation Rate #2 as the percentage of respondents who completed the survey of the eligible respondents who accessed the survey link, was 64.2% in 2018, 62.9% in 2019, 62.0% in 2020, 60.8% in 2021, 60.7% in 2022, and 63.9% in 2023 (American Association for Public Opinion Research 2016). Surveys were conducted in English or French. Median survey time was 20 min in 2018, 25 min in 2019, 21 min in 2020, 22 min in 2021, 23 min in 2022, and 23 min in 2023.

See ICPS Technical Reports for additional methodological details (Goodman and Hammond 2018; Goodman et al. 2019, 2020; Corsetti et al. 2021, 2022; Iraniparast et al. 2023).

### Measures

#### Sociodemographic characteristics

Respondents provided demographic information, including their country of residence, age, race/ethnicity, highest-level of education, and income adequacy (i.e. how difficult/easy it is for their family to make ends meet).

#### Gender identity

All respondents were asked to report their gender identity, with moderate differences in the survey questions across waves, as shown in Supplemental Table S1. Responses were aggregated into a derived gender identity variable (Cisgender women; Cisgender men; Transgender women; Transgender men; Gender-diverse; Unstated). Note that the ‘Cisgender women’ and ‘Cisgender men’ categories may not be cisgender-exclusive due to some of the question phrasing (described below). In Waves 1–5, sex assigned at birth and gender identity were used to categorize respondents as transgender women and transgender men. In Wave 6, respondents who selected any gender-diverse identity were categorized as gender-diverse. Respondents who indicated a transgender woman or transgender man identity without also indicating a gender-diverse identity were categorized as transgender women or transgender men, respectively. The gender identity of respondents who provided open-ended responses unrelated to gender identity were re-assigned to align with their sex assigned at birth in all waves. For a complete overview of gender identity categorization paths across all waves, see Supplemental Table S2.

#### Cannabis use frequency

Respondents’ frequency of cannabis consumption was categorized as ‘Non-consumer’, ‘Monthly/weekly consumer’, ‘Daily/almost daily consumer’. This was derived from responses to the questions “Have you ever tried marijuana?” (Yes; No), “How often do you use marijuana?” (Less than once per month; One or more times per month; One or more times per week; Every day or almost every day), and “When was the last time you used marijuana?” (More than 12 months ago; More than 3 months ago but less than 12 months ago; More than 30 days ago but less than 3 months ago; Within the past 30 days). Two derived variables were created: one to quantify respondents’ past 30-day cannabis use (Monthly consumer; Non-monthly consumer) and another to quantify respondents’ daily cannabis use (Daily/almost daily consumer; Non-daily consumer).

#### Reasons for cannabis use

A subsample of respondents (*n* = 53,297) who consumed cannabis in the past 12-months reported if their cannabis use was for medical and/or recreational purposes, due to differences in question wording across survey waves. Half of these respondents in Waves 3, 4, and 5 were asked “Do you self-identify as a medical marijuana user?” with response options of ‘Yes’, ‘No’, ‘Don’t know’, and ‘Refuse’. The other half of the Wave 5 respondents and all the Wave 6 respondents who consumed cannabis in the past 12-months were asked “Do you use marijuana for medical reasons, ‘recreational’ reasons, or both?” with response options of ‘Medical use only’, ‘Recreational use only’, ‘Both recreational and medical use’, ‘Don’t know’, ‘Refuse’. A derived variable was created to aggregate these responses (Use of medical cannabis; Exclusive recreational cannabis use).

#### Perceived addiction

Measures of one’s perceived addiction have been widely used as an indicator of dependence for tobacco and nicotine, as well as cannabis products (Chaiton et al. 2017; Vogel et al. 2019; Rundle 2024). Past 12-month cannabis consumers were asked “Do you consider yourself addicted to marijuana?” with response options of ‘Not at all addicted’, ‘A little addicted’, ‘Very addicted’, ‘Don’t know’, and ‘Refuse’.

#### Personal effects of cannabis use

Participants were asked to report positive and negative effects of cannabis using measures adapted from the Canadian Cannabis Survey, Health Canada’s national survey which measures cannabis consumers’ patterns of use and perceptions (Canada and Survey 2018). Past 12-month cannabis consumers were asked “During the past 12-months, what effect did your marijuana use have on your…”, with separate questions for the following seven domains: ‘Friendships or social life’, ‘Physical health’, ‘Mental health’, ‘Family life’, ‘Work’, ‘Studies’, and ‘Quality of life’. Each question had response options of ‘Negative effect’, ‘No effect’, ‘Positive effect’, ‘Not applicable’, ‘Don’t know’, and ‘Refuse’. The categorical responses for each domain were compiled into a continuous Personal Impacts index variable from 0–14, where greater scores indicate a more positive effect from consuming cannabis and near zero scores indicate negative effects from consuming cannabis. The Personal Impacts index score was calculated by adding the individual categorical responses for each domain as follows: 2 = ‘Positive effect’, 1 = ‘No effect’/‘Don’t know’, and 0 = ‘Negative effect’. ‘Not applicable’, and ‘Refuse’ responses were treated as missing data.

#### Comfort consuming cannabis

To assess an indicator of social norms using a measure which has been previously published in cannabis use research (Winfield-Ward and Hammond 2024), respondents were asked “How comfortable or uncomfortable would you feel openly using marijuana…” with separate questions for the following domains: ‘Around your parents’, ‘Around your boyfriend/girlfriend/partner/spouse’, ‘Around your friends’, ‘Around your co-workers’, ‘Around children’, and ‘In public’. Each question had response options of ‘Very uncomfortable’, ‘Uncomfortable’, ‘Neither’, ‘Comfortable’, ‘Very uncomfortable’, ‘Not applicable’, ‘Don’t know’, and ‘Refuse’. Responses were compiled into a Social Norms index variable from 0–6, where greater scores indicate greater levels of comfort openly consuming cannabis and near zero scores indicate low levels of comfort openly consuming cannabis. The Social Norms index score was categorized as follows: 1 = ‘Comfortable’/‘Very comfortable’ and 0 = ‘Uncomfortable’/‘Very uncomfortable’/‘Neither’/‘Don’t know’. ‘Not applicable’, and ‘Refuse’ responses were treated as missing data.

### Analysis

A total of 281 533 respondents completed the 2018–2023 ICPS surveys in Canada and the US. For all outcomes, sample exclusions were based on listwise deletion, including reasons for cannabis use (2 900 excluded), perceived addiction (647 excluded), personal impacts (3 627 excluded), and social norms (23 105 excluded).

Post-stratification sample weights were constructed based on known population targets for age, sex, education, region, race/ethnicity, and current smoking trends. A raking algorithm was applied to the cross-sectional analytic sample for each jurisdiction. Estimates are weighted unless otherwise specified.

Descriptive statistics were used to represent each outcome by gender identity. Binary logistic regression was used to model differences in past 30-day cannabis use and daily cannabis use; multinomial regression models examined differences in perceived addiction (0 = Not at all addicted, 1 = A little addicted, 2 = Very addicted); and linear regression was used to model the full range of differences in the personal impacts index (range 0 to 14) and social norms index (range 0 to 6). Each model controlled for age, race/ethnicity, education, and income adequacy as covariates that have been previously associated with cannabis use (National Academies of Sciences, Engineering, and Medicine 2024). Frequency of cannabis use was also included as a covariate in the latter three models. All pairwise comparisons were tested and reported in the tables. To support clarity, only the contrasts where cisgender men are the reference group are included in the figures.

## Results

### Sample characteristics

Table 1 shows the weighted and unweighted sample characteristics of respondents. Of the weighted sample, 48.8% of respondents identified as cisgender men, 48.9% of respondents identified as cisgender women, 0.4% identified as transgender men, 0.8% identified as transgender women, 0.2% had a broadly gender-diverse identity, and 1.0% had an unstated gender identity (see Table 1). Supplemental Table S3 shows sociodemographic characteristics by gender identity; notable differences were seen in country of residence (*p* = 0.024), age (*p* < 0.001), education (*p* < 0.001), race/ethnicity (*p* < 0.001), and income adequacy (*p* < 0.001). Overall, compared to the US participants, Canadians were less likely to identify as cisgender women or people with gender-diverse identities; transgender men, transgender women, and people with gender-diverse identities were younger and had lower educational attainment; transgender women and people with gender-diverse identities reported lower income adequacy; and people with unstated identities were less frequently white, and more frequently reported ‘unstated’ education and income.

**Table 1:** Sample characteristics, by country

	**Aggregated Sample**		**United States**		**Canada**	
	Weighted	Unweighted	Weighted	Unweighted	Weighted	Unweighted
	*n* = 281 533	*n* = 281 533	*n* = 187 621	*n* = 187 619	*n* = 93 912	*n* = 93 914
Gender identity						
Cisgender men	48.8% (137 220)	32.9% (92 664)	48.7% (91 310)	30.4% (57 005)	48.9% (45 740)	38.0% (35 659)
Cisgender women	48.9% (137 622)	65.1% (183 398)	49.0% (91 881)	67.6% (126 894)	48.7% (45 740)	60.2% (56 504)
Transgender men	0.4% (1 070)	0.4% (1 161)	0.4% (686)	0.4% (791)	0.4% (385)	0.4% (370)
Transgender women	0.8% (2 169)	0.5% (1 284)	0.8% (1 520)	0.5% (872)	0.7% (689)	0.4% (412)
Gender-diverse	0.2% (677)	0.3% (783)	0.2% (454)	0.3% (571)	0.2% (224)	0.2% (212)
Unstated	1.0% (2 776)	0.8% (2 243)	0.9% (1 771)	0.8% (1 486)	1.1% (1 005)	0.8% (757)
Age						
16–25	19.5% (54 853)	15.5% (43 511)	19.9% (37 385)	16.3% (30 548)	18.6% (17 468)	13.8% (12 963)
26–35	21.6% (60 831)	18.7% (52 621)	21.8% (40 917)	18.8% (35 242)	21.2% (19 914)	18.5% (17 379)
36–45	19.7% (55 600)	20.6% (57 982)	19.6% (36 681)	20.8% (38 946)	20.1% (18 919)	20.3% (19 036)
46–55	19.3% (54 344)	19.1% (53 824)	19.3% (36 139)	18.7% (35 050)	19.4% (18 205)	20.0% (18 774)
56–65	19.9% (55 906)	26.1% (73 595)	19.5% (36 500)	25.5% (47 833)	20.7% (19 406)	27.4% (25 762)
Education level						
Less than high school	11.6% (32 689)	8.3% (23 238)	10.1% (18 880)	8.4% (15 697)	14.7% (13 808)	8.0% (969)
High school diploma/equivalent	23.0% (64 825)	17.6% (49 578)	21.4% (40 162)	18.9% (35 481)	26.3% (24 663)	15.0% (7 541)
Some college/technical vocation	35.0% (98 441)	36.4% (102 570)	36.6% (68 662)	34.7% (35 043)	31.7% (29 779)	40.0% (14 097)
Bachelor’s degree/higher	29.5% (83 082)	36.9% (103 968)	31.2% (58 559)	37.4% (70 188)	26.1% (24 523)	36.0% (37 527)
Unstated	0.9% (2 496)	0.7% (2 179)	0.7% (1 358)	0.6% (1 210)	1.2% (1 139)	1.0% (33 780)
Race/ethnicity						
White	73.6% (207 256)	76.5% (215 407)	75.6% (141 756)	78.8% (147 756)	69.7% (65 499)	72.0% (67 651)
Other/Mixed/Unstated	26.4% (74 277)	23.5% (66 126)	24.4% (45 865)	21.3% (39 863)	30.3% (28 412)	28.0% (26 263)
Income adequacy						
Very easy/easy	31.3% (88 133)	31.5% (88 531)	31.6% (59 268)	31.3% (58 671)	30.7% (28 866)	31.8% (29 860)
Neither easy nor difficult	33.5% (94 302)	33.3% (93 851)	32.8% (61 462)	32.5% (30 947)	35.0% (32 840)	35.0% (32 904)
Very difficult/difficult	31.6% (88 987)	32.1% (90 281)	32.1% (60 299)	33.1% (62 057)	30.5% (28 687)	30.1% (28 224)
Unstated	3.6% (10 111)	3.2% (8 870)	3.5% (6 593)	3.1% (5 944)	3.7% (3 519)	3.1% (2 926)
Cannabis use						
Yearly	9.0% (25 336)	9.9% (27 879)	8.5% (15 988)	9.6% (17 952)	10.0% (9 349)	10.6% (9 927)
Monthly/weekly	12.2% (34 270)	11.5% (32 465)	12.0% (22 443)	11.5% (21 490)	12.6% (11 827)	11.7% (10 975)
Daily/almost daily	13.0% (36 552)	12.3% (34 514)	13.5% (25 362)	13.1% (24 607)	11.9% (11 190)	10.6% (9 907)
Non-consumer	65.8% (185 375)	66.3% (186 675)	66.0% (123 830)	65.9% (123 570)	65.5% (61 545)	67.2% (63 105)

### Cannabis use

#### Frequency of cannabis use

Overall, 25.2% of respondents reported consuming cannabis in the past 30 days. As seen in Table 2, there was no difference in past 30-day cannabis consumption between cisgender men, transgender men, transgender women, and people with gender-diverse identities. Based on further pairwise comparisons, cisgender women had lower odds of past 30-day cannabis use (22.0%) compared to all other groups, including cisgender men (28.1%), transgender men (32.7%), transgender women (27.0%), people with gender-diverse identities (28.4%), and people with unstated identities (30.4%). In addition, people with unstated identities had greater odds of past 30-day cannabis use, compared to cisgender men, transgender women, and people with gender-diverse identities. See Supplemental Table S4 for odds ratios, 95% confidence intervals, and p-levels for all covariates.

**Table 2:** Logistic regression model examining past 30-day cannabis use^a^ (*n* = 281 533)

		**Odds of past 30-day cannabis consumption**	
	**% Past 30-day consumers**^**b**^	**AOR (95%CI)**	**P level**
Cisgender men	**28.1%**	**Reference**	**Reference**
Cisgender women	22.0%	0.68 (0.66–0.70)	<.001
Transgender men	32.7%	1.12 (0.94–1.34)	.192
Transgender women	27.0%	0.88 (0.73–1.05)	.163
Gender-diverse	28.4%	0.88 (0.71–1.10)	.258
Unstated	30.4%	1.31 (1.13–1.52)	<.001
Cisgender women	**22.0%**	**Reference**	**Reference**
Cisgender men	28.1%	1.47 (1.43–1.51)	<.001
Transgender men	32.7%	1.65 (1.38–1.96)	<.001
Transgender women	27.0%	1.29 (1.07–1.55)	.007
Gender-diverse	28.4%	1.30 (1.04–1.61)	.018
Unstated	30.4%	1.92 (1.66–2.23)	<.001
Transgender men	**32.7%**	**Reference**	**Reference**
Cisgender men	28.1%	0.89 (0.75–1.06)	.192
Cisgender women	22.0%	0.61 (0.51–0.72)	<.001
Transgender women	27.0%	0.78 (0.61–1.00)	.054
Gender-diverse	28.4%	0.79 (0.60–1.04)	.086
Unstated	30.4%	1.16 (0.93–1.46)	.186
Transgender women	**27.0%**	**Reference**	**Reference**
Cisgender men	28.1%	1.14 (0.95–1.37)	.163
Cisgender women	22.0%	0.78 (0.65–0.93)	.007
Transgender men	32.7%	1.28 (1.00–1.65)	.054
Gender-diverse	28.4%	1.01 (0.76–1.33)	.963
Unstated	30.4%	1.49 (1.18–1.89)	.001
Gender-diverse	**28.4%**	**Reference**	**Reference**
Cisgender men	28.1%	1.13 (0.91–1.40)	.258
Cisgender women	22.0%	0.77 (0.62–0.96)	.018
Transgender men	32.7%	1.27 (0.97–1.67)	.086
Transgender women	27.0%	0.99 (0.75–1.31)	.963
Unstated	30.4%	1.48 (1.14–1.92)	.003
Unstated	**30.4%**	**Reference**	**Reference**
Cisgender men	28.1%	0.76 (0.66–0.89)	<.001
Cisgender women	22.0%	0.52 (0.45–0.60)	<.001
Transgender men	32.7%	0.86 (0.68–1.08)	.186
Transgender women	27.0%	0.67 (0.53–0.85)	.001
Gender-diverse	28.4%	0.67 (0.52–0.87)	.003

^a^Adjusted for country, age, education level, race/ethnicity, and income adequacy. See Supplemental Table S4 for full model output, including covariates

^b^Unadjusted percentage

The bolded gender identity indicates the reference group for the set of pairwise contrasts listed below

Overall, 13.0% of respondents reported daily cannabis use. As shown in Table 3, compared to cisgender men (14.1%), cisgender women had lower odds of daily cannabis use (11.7%), as did transgender women at 12.2%. People with unstated identities had the greatest odds of daily cannabis use (20.3%), compared to cisgender men, cisgender women, transgender men (14.2%), transgender women, and people with gender-diverse identities (15.4%). See Supplemental Table S5 for odds ratios, 95% confidence intervals, and p-levels for all covariates.

**Table 3:** Logistic regression model examining daily cannabis use^a^ (*n* = 281 533)

		**Odds of daily cannabis consumption**	
	**% Daily consumers**^**b**^	**AOR (95%CI)**	**P level**
**Cisgender men**	**14.1%**	**Reference**	**Reference**
Cisgender women	11.7%	0.74 (0.72–0.77)	<.001
Transgender men	14.2%	0.89 (0.72–1.11)	.295
Transgender women	12.2%	0.76 (0.59–1.00)	.047
Gender-diverse	15.4%	0.96 (0.73–1.26)	.760
Unstated	20.3%	1.63 (1.37–1.94)	<.001
Cisgender women	**11.7%**	**Reference**	**Reference**
Cisgender men	14.1%	1.35 (1.30–1.40)	<.001
Transgender men	14.2%	1.20 (0.97–1.49)	.093
Transgender women	12.2%	1.03 (0.79–1.35)	.818
Gender-diverse	15.4%	1.30 (0.99–1.69)	.059
Unstated	20.3%	2.20 (1.85–2.61)	<.001
Transgender men	**14.2%**	**Reference**	**Reference**
Cisgender men	14.1%	1.12 (0.90–1.39)	.295
Cisgender women	11.7%	0.83 (0.67–1.03)	.093
Transgender women	12.2%	0.86 (0.61–1.21)	.376
Gender-diverse	15.4%	1.08 (0.76–1.52)	.674
Unstated	20.3%	1.81 (1.39–2.40)	<.001
Transgender women	**12.2%**	**Reference**	**Reference**
Cisgender men	14.1%	1.31 (1.00–1.71)	.047
Cisgender women	11.7%	0.97 (0.74–1.27)	.818
Transgender men	14.2%	1.17 (0.83–1.64)	.376
Gender-diverse	15.4%	1.26 (0.86–1.83)	.237
Unstated	20.3%	2.13 (1.56–2.92)	<.001
Gender-diverse	**15.4%**	**Reference**	**Reference**
Cisgender men	14.1%	1.04 (0.80–1.37)	.760
Cisgender women	11.7%	0.77 (0.59–1.01)	.059
Transgender men	14.2%	0.93 (0.66–1.31)	.674
Transgender women	12.2%	0.80 (0.55–1.61)	.237
Unstated	20.3%	1.70 (1.24–2.33)	.001
Unstated	**20.3%**	**Reference**	**Reference**
Cisgender men	14.1%	0.61 (0.52–0.73)	<.001
Cisgender women	11.7%	0.45 (0.38–0.54)	<.001
Transgender men	14.2%	0.55 (0.42–0.72)	<.001
Transgender women	12.2%	0.47 (0.34–0.64)	<.001
Gender-diverse	15.4%	0.59 (0.43–0.81)	.001

^a^Adjusted for country, age, education level, race/ethnicity, and income adequacy. See Supplemental Table S5 for full model output, including covariates

^b^Unadjusted percentage

The bolded gender identity indicates the reference group for the set of pairwise contrasts listed below

### Using cannabis for medical reasons

**Fig. 1:**
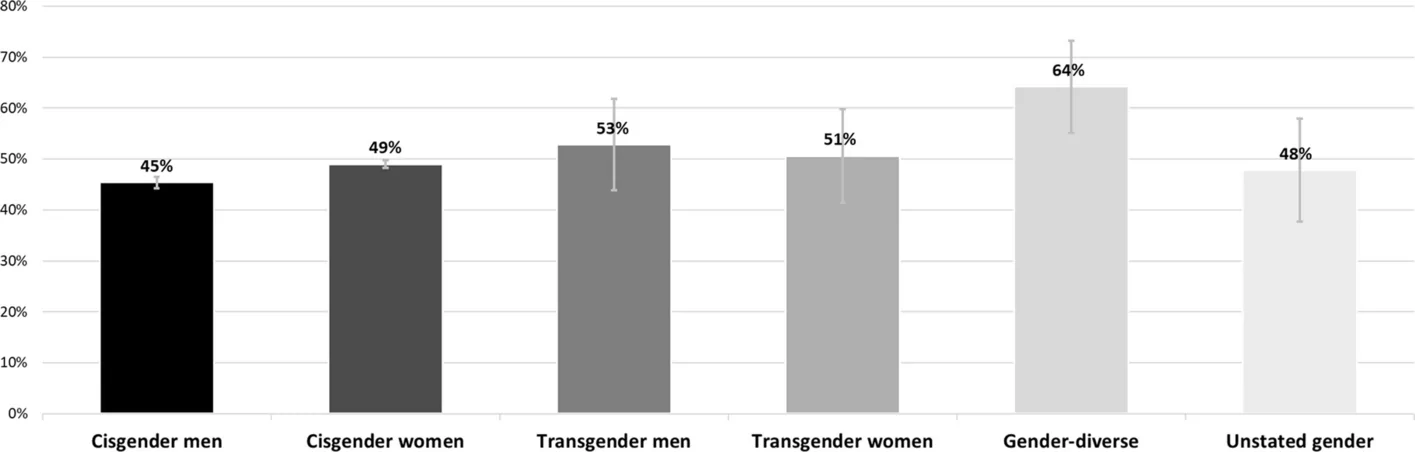
Cannabis consumption for medical reasons, among past 12-month consumers (*n* = 53 297). Consumption was highest among people with gender-diverse identities (64%), followed by transgender men (53%), transgender women (51%), cisgender women (49%), people with unstated identities (48%), and lowest among cisgender men (45%)

Overall, a total of 47.2% of past 12-month consumers reported using cannabis either for exclusively medical or both medical and recreational reasons. As shown in Fig. 1, use of cannabis for medical reasons was greatest among people with gender-diverse identities (64.2%) and lowest among cisgender men (45.4%) in 2020–2023 (see Supplemental Table S6).

### Perceived addiction to cannabis

**Fig. 2:**
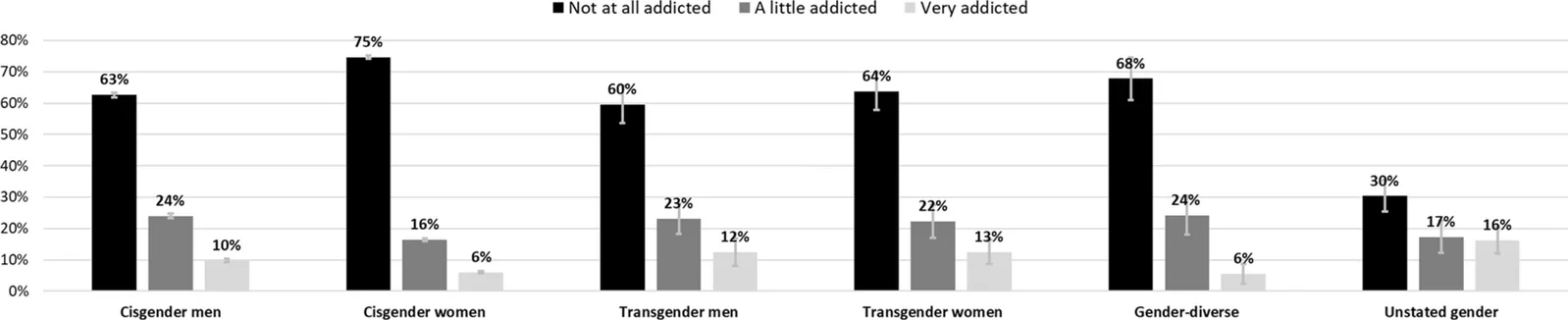
Perceived addiction to cannabis by gender identity, among past 12-month consumers (*n* = 94 211). Reports of being ‘a little addicted’ to cannabis were greatest among cisgender men and people with gender-diverse identities (24%), followed by transgender men (23%) and transgender women (22%), and lowest among people with unstated identities (17%) and cisgender women (16%). Reports of being ‘very addicted’ to cannabis were greatest among people with unstated identities (16%), followed by transgender women (13%), transgender men (12%) and cisgender men (10%), and lowest among cisgender women and people with gender-diverse identities (6%)

Overall, approximately 28.6% of past 12-month consumers self-reported any level of addiction to cannabis; less cisgender women reported being ‘very addicted’ and ‘a little addicted’ and more people with unstated identities reported ‘don’t know’ compared to all other identities. As shown in Fig. 2, for all gender identities other than unstated, most past 12-month cannabis consumers reported being ‘not at all addicted’ to cannabis.

There was no difference in the odds of reporting being ‘very addicted’ and being ‘a little addicted’ to cannabis between cisgender men (9.8%, 24.0%), transgender men (12.4%, 23.1%), and transgender women (12.5%, 22.2%). Cisgender women had lower odds of reporting being ‘very addicted’ (6.0%) and ‘a little addicted’ (16.4%) to cannabis compared to cisgender men, transgender men, and transgender women. People with gender-diverse identities had lower odds of reporting ‘very addicted’ (5.5%), compared to cisgender men, transgender men, and transgender women, but no difference in reporting ‘a little addicted’ (24.2%). People with unstated identities had greater odds of reporting ‘very addicted’ (16.2%) compared to cisgender men, cisgender women, transgender men, transgender women, and people with gender-diverse identities, in addition to greater odds of reporting ‘a little addicted’ (17.3%) compared to cisgender women and people with gender diverse identities. See Table 4 for further regression results. See Supplemental Table S7 for odds ratios, 95% confidence intervals, and p-levels for all covariates.

**Table 4:** Logistic regression model examining self-reported perceived addiction to cannabis among past 12-month consumers^a^ (*n* = 94 211)^b^

		**Odds of very addicted**^**d**^			**Odds of a little addicted**^**d**^			**Odds of don’t know**^**d**^	
	**% Very addicted**^**c**^	**AOR (95%CI)**	**P level**	**% A little addicted**^**c**^	**AOR (95%CI)**	**P level**	**% Don’t know**^**c**^	**AOR (95%CI)**	**P level**
Cisgender men	**9.8%**	**Reference**	**Reference**	**24.0%**	**Reference**	**Reference**	**3.6%**	**Reference**	**Reference**
Cisgender women	6.0%	0.57 (0.53–0.62)	<.001	16.4%	0.61 (0.58–0.64)	<.001	3.0%	0.73 (0.65–0.82)	<.001
Transgender men	12.4%	1.32 (0.84–2.08)	.232	23.1%	0.94 (0.69–1.26)	.656	5.0%	1.29 (0.67–2.47)	.445
Transgender women	12.5%	1.32 (0.87–1.99)	.190	22.2%	0.86 (0.61–1.21)	.391	1.6%	0.40 (0.19–0.83)	.013
Gender-diverse	5.5%	0.47 (0.24–0.91)	.025	24.2%	0.79 (0.56–1.12)	.187	2.5%	0.53 (0.19–1.51)	.237
Unstated	16.2%	3.27 (2.17–4.94)	<.001	17.3%	1.43 (0.96–2.11)	.077	36.2%	5.77 (3.86–8.63)	<.001
Cisgender women	**6.0%**	**Reference**	**Reference**	**16.4%**	**Reference**	**Reference**	**3.0%**	**Reference**	**Reference**
Cisgender men	9.8%	1.76 (1.62–1.90)	<.001	24.0%	1.64 (1.56–1.73)	<.001	3.6%	1.37 (1.23–1.54)	<.001
Transgender men	12.4%	2.32 (1.47–3.64)	<.001	23.1%	1.54 (1.14–2.06)	.005	5.0%	1.77 (0.93–2.28)	.084
Transgender women	12.5%	2.32 (1.52–3.50)	<.001	22.2%	1.41 (1.01–1.99)	.047	1.6%	0.55 (0.27–1.13)	.104
Gender-diverse	5.5%	0.82 (0.42–1.60)	.560	24.2%	1.30 (0.92–1.84)	.140	2.5%	0.73 (0.26–2.07)	.555
Unstated	16.2%	5.75 (3.81–8.67)	<.001	17.3%	2.34 (1.58–3.46)	<.001	36.2%	7.92 (5.32–11.81)	<.001
Transgender men	**12.4%**	**Reference**	**Reference**	**23.1%**	**Reference**	**Reference**	**5.0%**	**Reference**	**Reference**
Cisgender men	9.8%	0.76 (0.48–1.19)	.232	24.0%	1.07 (0.80–1.44)	.656	3.6%	0.78 (0.41–1.49)	.445
Cisgender women	6.0%	0.43 (0.28–0.68)	.001	16.4%	0.65 (0.48–0.88)	.005	3.0%	0.57 (0.30–1.08)	.084
Transgender women	12.5%	1.00 (0.55–1.83)	.999	22.2%	0.92 (0.59–1.44)	.720	1.6%	0.31 (0.12–0.81)	.017
Gender-diverse	5.5%	0.35 (0.16–0.79)	.011	24.2%	0.85 (0.54–1.33)	.469	2.5%	0.41 (0.12–1.40)	.156
Unstated	16.2%	2.48 (1.36–4.54)	.003	17.3%	1.53 (0.94–2.49)	.091	36.2%	4.48 (2.11–9.51)	<.001
Transgender women	**12.5%**	**Reference**	**Reference**	**22.2%**	**Reference**	**Reference**	**1.6%**	**Reference**	**Reference**
Cisgender men	9.8%	0.76 (0.50–1.15)	.190	24.0%	1.16 (0.83–1.64)	.391	3.6%	2.50 (1.21–5.17)	.013
Cisgender women	6.0%	0.43 (0.29–0.65)	<.001	16.4%	0.71 (0.50–1.00)	.047	3.0%	1.82 (0.88–3.75)	.104
Transgender men	12.4%	1.00 (0.55–1.83)	.999	23.1%	1.09 (0.69–1.70)	.720	5.0%	3.22 (1.23–8.44)	.017
Gender-diverse	5.5%	0.36 (0.16–0.77)	.009	24.2%	0.92 (0.57–1.49)	.731	2.5%	1.33 (0.38–4.70)	.657
Unstated	16.2%	2.48 (1.40–4.42)	.002	17.3%	1.66 (0.99–2.78)	.056	36.2%	14.44 (6.34–32.89)	<.001
Gender-diverse	**5.5%**	**Reference**	**Reference**	**24.2%**	**Reference**	**Reference**	**2.5%**	**Reference**	**Reference**
Cisgender men	9.8%	2.14 (1.10–4.16)	.025	24.0%	1.27 (0.89–1.79)	.187	3.6%	1.88 (0.66–5.34)	.237
Cisgender women	6.0%	1.22 (0.63–2.37)	.560	16.4%	0.77 (0.54–1.09)	.140	3.0%	1.37 (0.48–3.88)	.555
Transgender men	12.4%	2.82 (1.27–6.27)	.011	23.1%	1.18 (0.75–1.86)	.469	5.0%	2.42 (0.71–8.20)	.156
Transgender women	12.5%	2.82 (1.30–6.13)	.009	22.2%	1.09 (0.67–1.77)	.731	1.6%	0.75 (0.21–2.66)	.657
Unstated	16.2%	7.00 (3.22–15.24)	<.001	17.3%	1.80 (1.07–3.04)	.027	36.2%	10.85 (3.54–33.25)	<.001
Unstated	**16.2%**	**Reference**	**Reference**	**17.3%**	**Reference**	**Reference**	**36.2%**	**Reference**	**Reference**
Cisgender men	9.8%	0.31 (0.20–0.46)	<.001	24.0%	0.70 (0.47–1.04)	.077	3.6%	1.17 (0.12–0.26)	<.001
Cisgender women	6.0%	0.17 (0.12–0.26)	<.001	16.4%	0.43 (0.29–0.63)	<.001	3.0%	0.13 (0.09–0.19)	<.001
Transgender men	12.4%	0.40 (0.22–0.74)	.003	23.1%	0.66 (0.40–1.07)	.091	5.0%	0.22 (0.11–0.47)	<.001
Transgender women	12.5%	0.40 (0.23–0.72)	.002	22.2%	0.60 (0.36–1.01)	.056	1.6%	0.07 (0.03–0.16)	<.001
Gender-diverse	5.5%	0.14 (0.07–0.31)	<.001	24.2%	0.56 (0.33–0.93)	.027	2.5%	0.09 (0.03–0.28)	<.001

^a^Adjusted for country, age, education level, race/ethnicity, and income adequacy. See Supplemental Table S7 for full model output, including covariates

^b^647 dropped due to refused

^c^Unadjusted percentage

^d^ ‘Not at all addicted’ is the reference group

The bolded gender identity indicates the reference group for the set of pairwise contrasts listed below

### Personal effects of cannabis use

**Fig. 3:**
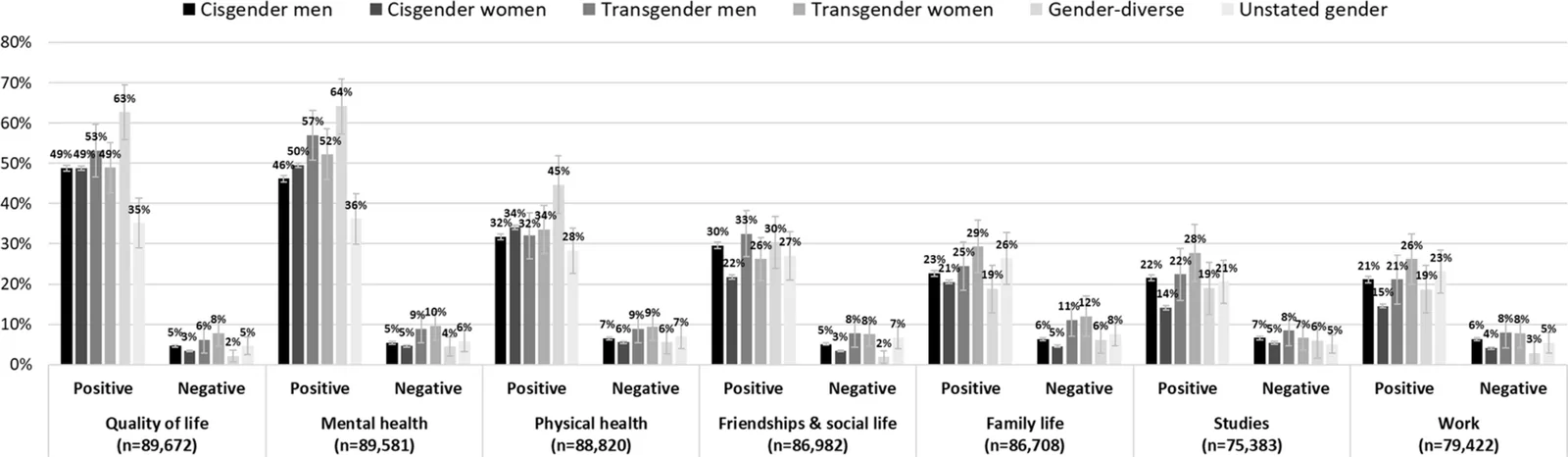
Personal effects of cannabis use by gender identity, among past 12-month consumers. Negative effects were outweighed by positive effects across all domains and gender identities. People with gender-diverse identities reported the greatest positive effects on quality of life (63%), mental health (64%), and physical health (45%). Less people with unstated identities reported positive effects on quality of life (35%), mental health (36%), and physical health (28%) compared to other identities. Less cisgender women reported positive effects on friendships and social life (22%), studies (14%), and at work (15%), compared to other identities. Transgender men and transgender women consistently reported the greatest negative effects across domains

Figure 3 shows a summary of gender differences for each personal effect from cannabis use, individually. Generally, reports of positive effects on respondents’ quality of life, mental health, physical health, friendships and social life, family life, studies, and work were consistent across gender identities; with the exception of greater positive effects on physical health, mental health, and quality of life among people with gender-diverse identities (see Supplemental Table S8).

Overall, the mean score on the personal impacts index (range 0–14) was 8.11 among past 12-month cannabis consumers. Table 5 illustrates that there was no difference in the mean reports of the personal effects of cannabis use among cisgender men (mean = 8.16), cisgender women (mean = 8.06), transgender men (mean = 8.15), and transgender women (mean = 8.21) who consumed cannabis in the past 12-months. With a mean index score of 8.93, people with gender-diverse identities reported greater positive personal effects from cannabis use than cisgender men, cisgender women, transgender men, and people with unstated identities. With a mean index score of 7.30, people with unstated identities reported fewer positive personal effects from cannabis use than cisgender men, cisgender women, and transgender women. See Supplemental Table S9 for odds ratios, 95% confidence intervals, and p-levels for all covariates.

**Table 5:** Linear regression model examining personal impacts of cannabis use among past 12-month consumers^a^ (*n* = 91 231)^b^

	**Mean (± Std Error)**^**c**^	**β (95%CI)**	**P level**
Cisgender men	**8.16 (± 0.02)**	**Reference**	**Reference**
Cisgender women	8.06 (± 0.02)	−0.01 ([−0.07]−0.04)	.671
Transgender men	8.15 (± 0.20)	−0.02 ([−0.39]−0.35)	.916
Transgender women	8.21 (± 0.19)	0.11 ([−0.25]−0.46)	.563
Gender-diverse	8.63 (± 0.17)	0.53 (0.21–0.86)	.001
Unstated	7.30 (± 0.20)	−0.50 ([−0.91]-[−0.10])	.014
Cisgender women	**8.06 (± 0.02)**	**Reference**	**Reference**
Cisgender men	8.16 (± 0.02)	0.01 ([−0.04]−0.07)	.671
Transgender men	8.15 (± 0.20)	−0.01 ([−0.37]−0.36)	.966
Transgender women	8.21 (± 0.19)	0.12 ([−0.24]−0.47)	.518
Gender-diverse	8.63 (± 0.17)	0.54 (0.22–0.87)	.001
Unstated	7.30 (± 0.20)	−0.49 ([−0.89]-[−0.09])	.016
Transgender men	**8.15 (± 0.20)**	**Reference**	**Reference**
Cisgender men	8.16 (± 0.02)	0.02 ([−0.35]−0.39)	.916
Cisgender women	8.06 (± 0.02)	0.01 ([−0.36]−0.37)	.966
Transgender women	8.21 (± 0.19)	0.13 ([−0.38]−0.63)	.627
Gender-diverse	8.63 (± 0.17)	0.55 (0.07–1.04)	.025
Unstated	7.30 (± 0.20)	−0.48 ([-1.02]-0.05)	.078
Transgender women	**8.21 (± 0.19)**	**Reference**	**Reference**
Cisgender men	8.16 (± 0.02)	−0.11 ([−0.46]−0.25)	.563
Cisgender women	8.06 (± 0.02)	−0.12 ([−0.47]−0.24)	.518
Transgender men	8.15 (± 0.20)	−0.13 ([−0.63]−0.38)	.627
Gender-diverse	8.63 (± 0.17)	0.43 ([−0.05]−0.91)	.080
Unstated	7.30 (± 0.20)	−0.61 ([−1.15]-[−0.07])	.026
Gender-diverse	**8.63 (± 0.17)**	**Reference**	**Reference**
Cisgender men	8.16 (± 0.02)	−0.53 ([−0.86]-[-0.21])	.001
Cisgender women	8.06 (± 0.02)	−0.54 ([−0.87]-[−0.22])	.001
Transgender men	8.15 (± 0.20)	−0.55 ([−1.04]-[−0.07])	.025
Transgender women	8.21 (± 0.19)	−0.43 ([−0.91]-0.05)	.080
Unstated	7.30 (± 0.20)	−1.04 ([−1.55]-[-0.52])	<.001
Unstated	**7.30 (± 0.20)**	**Reference**	**Reference**
Cisgender men	8.16 (± 0.02)	0.50 (0.10–0.91)	.014
Cisgender women	8.06 (± 0.02)	0.49 (0.09–0.89)	.016
Transgender men	8.15 (± 0.20)	0.48 ([−0.05]−1.02)	.078
Transgender women	8.21 (± 0.19)	0.61 (0.07–1.15)	.026
Gender-diverse	8.63 (± 0.17)	1.04 (0.52–1.55)	<.001

^a^Adjusted for country, age, education level, race/ethnicity, and income adequacy. See Supplemental Table S9 for full model output, including covariates

^b^Respondents dropped due to ‘refused’ or ‘not applicable’ for all items included in the index

^c^Unadjusted mean comes from the index from 0–14 of all seven of the personal effect questions

The bolded gender identity indicates the reference group for the set of pairwise contrasts listed below

### Comfort consuming cannabis in social settings

**Fig. 4:**
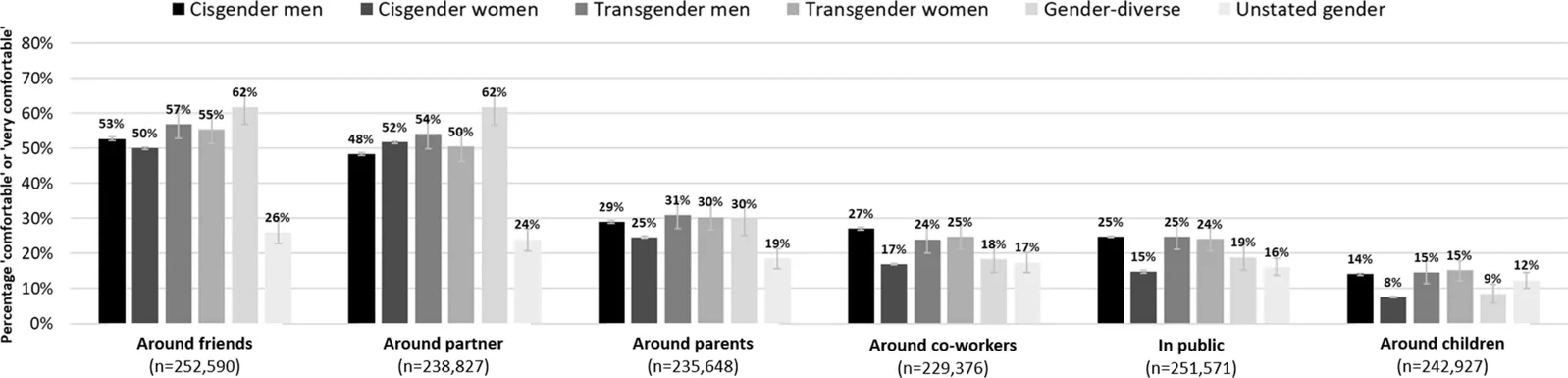
Comfort consuming cannabis in social settings by gender identity, among all respondents. Comfort was generally similar between identities across social settings. Comfort was greatest around friends and partners, followed by parents, co-workers, and in public, with the least comfort reported among children. Less people with unstated identities reported being ‘comfortable’ or ‘very comfortable’ around friends (26%), partners (24%), and parents (19%). Comfort consuming cannabis in public was lowest among cisgender women (15%) and people with unstated identities (16%), and comfort consuming cannabis around children was lowest among cisgender women (8%) and people with gender-diverse identities (9%)

Figure 4 shows a summary of gender differences in comfort consuming cannabis in each social setting, individually. Generally, people with unstated identities and cisgender women reported low comfort consuming cannabis around friends, partners, parents, the public, and children. Greater comfort was reported among cisgender men, transgender men, and transgender women, and comfort fluctuated among people with gender-diverse identities (see Supplemental Table S10).

Overall, the mean score on the social norms index (range 0–6) was 1.70 among all respondents. As shown in Table 6 there was no difference in the mean level of comfort consuming cannabis across each of the social settings among cisgender men (mean = 1.85), transgender men (mean = 1.94), transgender women (mean = 1.88), and people with gender-diverse identities (mean = 1.84). With a mean score of 1.55, cisgender women felt less comfort compared to cisgender men, transgender men, transgender women, and people with gender-diverse identities. With a mean score of 1.06, people with unstated identities reported the least comfort compared to cisgender men, cisgender women, transgender men, transgender women, and people with gender-diverse identities. See Supplemental Table S11 for odds ratios, 95% confidence intervals, and p-levels for all covariates.

**Table 6:** Linear regression model examining social norms via comfort consuming cannabis, among all respondents^a^ (*n* = 258 428)^b^

	**Mean (± Std Error)**^**c**^	**β (95%CI)**	**P level**
Cisgender men	**1.85 (± 0.01)**	**Reference**	**Reference**
Cisgender women	1.55 (± 0.01)	−0.21 ([−0.23]-[−0.19])	<.001
Transgender men	1.94 (± 0.08)	0.03 ([−0.11]−0.17)	.668
Transgender women	1.88 (± 0.07)	0.07 ([−0.06]−0.19)	.282
Gender-diverse	1.84 (± 0.08)	−0.05 ([−0.18]-0.09)	.518
Unstated	1.06 (± 0.06)	−0.43 ([−0.54]-[−0.32])	<.001
Cisgender women	**1.55 (± 0.01)**	**Reference**	**Reference**
Cisgender men	1.85 (± 0.01)	0.21 (0.19–0.23)	<.001
Transgender men	1.94 (± 0.08)	0.24 (0.10–0.38)	.001
Transgender women	1.88 (± 0.07)	0.28 (0.16–0.40)	<.001
Gender-diverse	1.84 (± 0.08)	0.17 (0.03–0.30)	.017
Unstated	1.06 (± 0.06)	−0.22 ([-0.33]-[-0.11])	<.001
Transgender men	**1.94 (± 0.08)**	**Reference**	**Reference**
Cisgender men	1.85 (± 0.01)	−0.03 ([−0.17]−0.11)	.668
Cisgender women	1.55 (± 0.01)	−0.24 ([−0.38]-[−0.10])	.001
Transgender women	1.88 (± 0.07)	0.04 ([−0.15]−0.22)	.691
Gender-diverse	1.84 (± 0.08)	−0.08 ([−0.27]−0.12)	.443
Unstated	1.06 (± 0.06)	−0.46 ([−0.64]-[-0.28])	<.001
Transgender women	**1.88 (± 0.07)**	**Reference**	**Reference**
Cisgender men	1.85 (± 0.01)	−0.07 ([−0.19]−0.06)	.282
Cisgender women	1.55 (± 0.01)	−0.28 ([−0.40]-[−0.16])	<.001
Transgender men	1.94 (± 0.08)	−0.04 ([−0.22]−0.15)	.691
Gender-diverse	1.84 (± 0.08)	−0.11 ([−0.30]−0.07)	.224
Unstated	1.06 (± 0.06)	−0.50 ([−0.66]-[−0.33])	<.001
Gender-diverse	**1.84 (± 0.08)**	**Reference**	**Reference**
Cisgender men	1.85 (± 0.01)	0.05 ([−0.09]−0.18)	.518
Cisgender women	1.55 (± 0.01)	−0.17 ([-0.30]−[-0.03])	.017
Transgender men	1.94 (± 0.08)	0.08 ([−0.12]−0.27)	.443
Transgender women	1.88 (± 0.07)	0.11 ([−0.07]−0.30)	.224
Unstated	1.06 (± 0.06)	-0.38 ([−0.26]-[−0.21])	<.001
Unstated	**1.06 (± 0.06)**	**Reference**	**Reference**
Cisgender men	1.85 (± 0.01)	0.43 (0.32–0.54)	<.001
Cisgender women	1.55 (± 0.01)	0.22 (0.11–0.33)	<.001
Transgender men	1.94 (± 0.08)	0.46 (0.28–0.64)	<.001
Transgender women	1.88 (± 0.07)	0.50 (0.33–0.66)	<.001
Gender-diverse	1.84 (± 0.08)	0.38 (0.21–0.56)	<.001

^a^Adjusted for country, age, education level, race/ethnicity, and income adequacy. See Supplemental Table S11 for full model output, including covariates

^b^Respondents dropped due to ‘refused’ or ‘not applicable’ for all items included in the index

^c^Unadjusted mean comes from the index from 0–6 of all six of the comfort consuming cannabis question

The bolded gender identity indicates the reference group for the set of pairwise contrasts listed below

## Discussion

The current study is among the first to examine the association between gender identity and cannabis at the population-level. Cisgender women consumed cannabis less frequently than all other gender identities, with the same proportion of transgender women reporting daily consumption; this is consistent with existing findings that cannabis consumption is least frequent among women (Hemsing and Greaves 2020; Greaves and Hemsing 2020; Matheson and Le Foll 2023). In general, cisgender men, transgender men, people with gender-diverse identities, and people with unstated identities reported similar frequencies of cannabis consumption. Almost one-third of transgender men, transgender women, and people with gender-diverse identities reported monthly cannabis consumption. This aligns with one scoping review’s estimate that 30% of people with transgender and gender-diverse identities consume cannabis monthly (Ruppert et al. 2021). However, the similarities in monthly and daily consumption between cisgender men and people with transgender and gender-diverse identities differ from previous findings that people with cisgender identities consume cannabis less frequently (Day et al. 2017; Christian et al. 2018). The limited sample size and inclusion of people with transgender and gender-diverse identities common to the literature may limit previous estimates and account for discrepancy in previous findings.

Trends in medical cannabis consumption were similar across identities. Approximately half of cisgender men, cisgender women, transgender men, transgender women, and people with unstated identities who consumed cannabis in the past 12-months reported using cannabis either exclusively for medical reasons or for both medical and recreational reasons. People with gender-diverse identities were the exception, with around two-thirds reporting any medical cannabis use. This novel finding that cannabis is commonly used as support for medical reasons across all gender identities suggests that current medical cannabis-related policies and stigmas do not deter access based on gender identity alone. However, the current findings only assessed ‘reasons’ for medical cannabis use, not formal authorization. Given that authorization typically requires interaction with a health care provider, for which there may be differential access among both gender groups and marginalized groups, future studies should examine formal authorization in jurisdictions where medical cannabis is legal.

Perceived addiction to cannabis was relatively high across all gender identities. Any addiction to cannabis was lowest among cisgender women who consumed cannabis in the past 12-months. Gender differences in addiction to cannabis have not been estimated in the literature; however, males and men consume cannabis more frequently and have a greater likelihood of developing CUD compared to females and women (Greaves and Hemsing 2020; Leung et al. 2020). These sex/gender differences are reflected in the notable differences in being ‘very’ or ‘a little’ addicted to cannabis between cisgender men (10%, 24%) and cisgender women (6%, 16%) in the current study. Generally, cisgender men, transgender men, transgender women, and people with gender-diverse identities self-reported greater proportions of addiction across all levels. The high rate of perceived addiction among people with transgender and gender-diverse identities in the current study is also consistent with reports that these groups are at the greatest risk of CUD, possibly due to experiences of marginalization (Dyar 2022).

Personal effects from cannabis reported by respondents who had consumed cannabis in the past 12-months were generally positive. Across all identities, reports of positive effects were greatest for quality of life and mental health, whereas reports of negative effects were greatest for physical health and family life. People with unstated identities had the lowest score on the personal impacts index. This is consistent with the other reports from people with unstated identities, which consistently fell on the extreme high- or low-ends of the responses. The exact reason that these respondents chose not to state their gender identities is unknown, which limits interpretation. People with gender-diverse identities had the highest score on the personal impacts index: approximately 10% more people with gender-diverse identities reported positive effects on quality of life, mental health, and physical health compared to other identities. There were no differences in scores on the personal impact index across other gender identities; however, differences were present within many of the effects. To our knowledge, no other study has assessed differences in the experience of these personal effects across gender identities. However, the positive effects on quality of life and mental health reported by people with transgender and gender-diverse identities may reflect the perceived benefits of using cannabis to express and understand one’s own gender, as well as to deal with the stress of gender-related discrimination (Hemsing and Greaves 2020; Barborini et al. 2024; London-Nadeau et al. 2025).

Comfort consuming cannabis across social settings was moderately low. Across all gender identities, reports of comfort were lowest around children, and greatest around friends and partners, as expected. Cisgender men, transgender men, and transgender women reported similar levels of comfort: approximately half reported comfort consuming cannabis around friends and partners, one-third reported comfort around parents, one-quarter reported comfort around co-workers and in public, and 15% reported comfort around children. Fewer cisgender women reported comfort consuming cannabis among co-workers, the public, and children. People with unstated identities scored the lowest on the social norms index, with reports of comfort around friends, partners, parents, and co-workers at least 10% lower than cisgender men, transgender men, and transgender women. People with gender-diverse identities’ reports of comfort fluctuated: they reported the greatest comfort consuming cannabis around friends and partners (62%), but low comfort around co-workers, the public, and children. These estimates are consistent with qualitative literature which has identified cannabis consumption as a means of expressing and understanding gender identity among people with transgender and gender-diverse identities (Hemsing and Greaves 2020; London-Nadeau et al. 2025). The current findings are also consistent with the general perception of cannabis consumption as ‘masculine’, and qualitative reports of cannabis consumption to express masculinity (Hemsing and Greaves 2020; Barborini et al. 2024). To our knowledge, estimates of comfort consuming cannabis by gender identity have not been previously identified.

### Limitations

There are several limitations to the current study. As respondents were recruited using non-probability-based sampling, findings may not be nationally representative. Data were weighted using age, sex, education, and smoking status to make estimates more representative of the Canadian and the US population. The phrasing of the gender identity question in the first five survey waves included either a broad ‘Transgender’ or ‘Other’ response option but did not include response options for ‘Transgender woman’ or ‘Transgender man’, specifically. To address this, data were recoded by cross-referencing gender identity with the sex on respondents’ birth certificates. Given that that respondents may have reported their gender identity as their sex assigned at birth or legally changed their sex on their birth certificate, respondents in the ‘Woman’ and ‘Man’ categories are not necessarily cisgender. Additionally, results were aggregated across survey waves and country due to the small sample size of people with transgender and gender-diverse identities. Consequently, potential differences related to time, the COVID-19 pandemic, and cannabis legalization were not examined. Furthermore, the personal nature of the questions explored in this study make the findings subject to social desirability bias. Reports of gender identity, perceived addiction, personal effects, and comfort consuming cannabis may skew toward options that respondents perceive as ‘normal’ or desirable.

## Conclusion

Cisgender men, transgender men, and transgender women reported modestly higher levels of cannabis use, perceived addiction, and comfort consuming cannabis compared to cisgender women. Transgender men and transgender women’s experiences of marginalization may partially explain these patterns. The ‘masculine’ perception of cannabis use may also contribute to these patterns among cisgender and transgender men, and reflect gendered social norms regarding the acceptability of cannabis consumption or comfort seeking help. Due to the increased health risks associated with frequent cannabis use and addiction, cannabis policies should consider gender-related disparities. This could include tailored messaging regarding safe consumption and health warnings that reflect both sex-related and gender-related factors linked to cannabis use, in addition to addressing barriers to receiving support for addiction. Future qualitative and quantitative research should seek to further understand the reasons for these differences by gender identity, in addition to building evidence focused on specific gender-diverse and gender minority populations (i.e., non-binary, gender-queer, Two-Spirit, etc.). This includes further exploring differences in relevant outcomes and indicators, including mental health and quality of life, across identities.

## Electronic supplementary material

Supplementary Material 1.

## Data Availability

The dataset analyzed during the current study is available from the corresponding author on reasonable request.

## Abbreviations

CUD: Cannabis Use Disorder
THC: Tetrahydrocannabinol
ICPS: International Cannabis Policy Study
