# Supplementary material for: Gender identity and cannabis use in Canada and the United States: cross-sectional analysis of the International Cannabis Policy Study
Source: J Cannabis Res. 2026 Jun 12;8:95. doi: 10.1186/s42238-026-00452-6 (PMC13501832; doi:10.1186/s42238-026-00452-6)
Supplement: Supplementary file 1 [file 42238_2026_452_MOESM1_ESM.docx]

**Supplemental Table S1: Gender identity question phrasing and response options across survey waves, 2018-2023**

| **Survey year** | **Question phrasing and response options** |
| --- | --- |
|  |  |
|  |  |
| **2018** | “How would you describe your gender identity today?”  [‘Female’; ‘Male’; ‘Transgender’; ‘Do not identify as female, male, or transgender’; ‘Other’; ‘Don’t know’; ‘Refuse’]. |
|  |  |
|  |  |
| **2019–2022** | “How would you describe your gender today?”  [‘Woman’; ‘Man’; ‘Other’; ‘Don’t know’; ‘Refuse’]. |
|  |  |
|  |  |
| **2023** | “What terms best express how you describe your gender identity? Select all that apply.”  [‘Man’; ‘Woman’; ‘Non-binary’; ‘Transgender’; ‘None of these describe me’; ‘Don’t know’; ‘Refuse’]  If selected ‘None of these describe me’:  [‘Woman’; ‘Man’; ‘Trans woman/Transgender Woman/MTF’; ‘Trans man/Transgender man/WTF’; ‘Nonbinary’; ‘Genderqueer’; ‘Genderfluid’; ‘An identity not listed here, please describe’; ‘Questioning or unsure’; ‘None of these describe me, and I want to specify’]. |
|  |  |

**Supplemental Table S2: Gender identity categorization paths for all response combinations across survey waves, 2018-2023**

| **Survey year** | **Gender identity response** | **Sex assigned at birth response** | **Current gender identity category** |
| --- | --- | --- | --- |
|  |  |  |  |
|  |  |  |  |
| **2018** |  |  |  |
|  | Female | Female | Cisgender woman |
|  | Male | Male | Cisgender man |
|  | Female | Male | Transgender woman |
|  | Transgender | Male | Transgender woman |
|  | Male | Female | Transgender man |
|  | Transgender | Female | Transgender man |
|  | Don’t identify as female, male, or transgender | - | Gender-diverse |
|  | Other | - | As described in open-ended response |
|  | Don’t know | - | Unstated |
|  | Refuse | - | Unstated |
|  |  |  |  |
| **2019-2022** |  |  |  |
|  | Woman | Female | Cisgender woman |
|  | Woman | Intersex | Cisgender woman |
|  | Man | Male | Cisgender man |
|  | Man | Intersex | Cisgender man |
|  | Woman | Male | Transgender woman |
|  | Man | Female | Transgender man |
|  | Other | - | As described in open-ended response |
|  | Don’t know | - | Unstated |
|  | Refuse | - | Unstated |
|  |  |  |  |
| **2023** |  |  |  |
|  | Woman | Female | Cisgender woman |
|  | Woman | Intersex | Cisgender woman |
|  | Man | Male | Cisgender man |
|  | Man | Intersex | Cisgender man |
|  | Trans woman/Transgender woman/MTF | - | Transgender woman |
|  | Transgender + Woman | - | Transgender woman |
|  | Transgender + Trans woman/Transgender woman/MTF | - | Transgender woman |
|  | Transgender | Male | Transgender woman |
|  | Woman | Male | Transgender woman |
|  | Trans man/Transgender man/FTM | - | Transgender man |
|  | Transgender + Man | - | Transgender man |
|  | Transgender + Trans man/Transgender man/FTM | - | Transgender man |
|  | Transgender | Female | Transgender man |
|  | Man | Female | Transgender man |
|  | Non-binary | - | Gender-diverse |
|  | Nonbinary | - | Gender-diverse |
|  | Genderqueer | - | Gender-diverse |
|  | Genderfluid | - | Gender-diverse |
|  | Questioning/unsure | - | Gender-diverse |
|  | Woman + Man | - | Gender-diverse |
|  | Nonbinary + Transgender | - | Gender-diverse |
|  | Nonbinary + None of these | - | Gender-diverse |
|  | An identity not listed here, please describe | - | As described in open-ended response |
|  | None of these describe me, and I want to specify | - | As described in open-ended response |
|  | Don’t know | - | Unstated |
|  | Refuse | - | Unstated |
|  |  |  |  |

**Supplemental Table S3: Sample characteristics, by gender identity (N=281 553)**

|  | **Cisgender men** | | **Cisgender women** | | **Transgender men** | | **Transgender women** | | **Gender-diverse** | | **Unstated** | |
| --- | --- | --- | --- | --- | --- | --- | --- | --- | --- | --- | --- | --- |
|  | Weighted | Unweighted | Weighted | Unweighted | Weighted | Unweighted | Weighted | Unweighted | Weighted | Unweighted | Weighted | Unweighted |
|  | n=92 664 | n=92 664 | n=183 398 | n=183 398 | n=1 161 | n=1 161 | n=1 284 | n=1 284 | n=783 | n=783 | n=2 243 | n=2 243 |
|  |  |  |  |  |  |  |  |  |  |  |  |  |
| **Country** |  |  |  |  |  |  |  |  |  |  |  |  |
| Canada | 33.5%  (45 909) | 38.5%  (35 659) | 33.2%  (45 740) | 30.8%  (56 504) | 35.9%  (385) | 31.9%  (370) | 29.9%  (649) | 32.1%  (412) | 33.0%  (224) | 27.1%  (212) | 36.2%  (1 005) | 33.8%  (757) |
|  |  |  |  |  |  |  |  |  |  |  |  |  |
| **Age** |  |  |  |  |  |  |  |  |  |  |  |  |
| 16-25 | 19.1%  (26 161) | 15.5%  (14 323) | 18.6%  (25 549) | 14.5%  (26 808) | 47.5%  (509) | 43.9%  (510) | 48.9%  (1 061) | 45.6%  (585) | 57.1%  (387) | 54.4%  (426) | 42.8%  (1 187) | 38.3%  (859) |
| 26-35 | 22.0%  (30 165) | 16.7%  (15 427) | 21.1%  (29 023) | 19.6%  (35 923) | 25.0%  (268) | 23.8%  (276) | 18.4%  (401) | 15.3%  (197) | 28.7%  (195) | 26.7%  (209) | 28.1%  (781) | 26.3%  (589) |
| 36-45 | 20.0%  (27 459) | 21.4%  (19 799) | 19.7%  (27 138) | 20.3%  (37 268) | 14.1%  (151) | 15.7%  (182) | 15.9%  (345) | 17.4%  (223) | 7.9%  (54) | 11.4%  (89) | 16.4%  (455) | 18.8%  (421) |
| 46-55 | 19.2%  (26 356) | 18.6%  (17 260) | 19.9%  (27 409) | 19.7%  (36 065) | 8.2%  (89) | 8.8%  (102) | 9.3%  (202) | 10.1%  (129) | 4.4%  (31) | 4.7%  (37) | 9.3%  (259) | 10.3%  (231) |
| 56-65 | 19.7%  (27 078) | 27.9%  (25 855) | 20.7%  (28 501) | 25.8%  (47 334) | 5.2%  (56) | 7.8%  (91) | 7.5%  (162) | 11.7%  (150) | 1.9%  (14) | 2.8%  (22) | 3.5%  (96) | 6.4%  (143) |
|  |  |  |  |  |  |  |  |  |  |  |  |  |
| **Education** |  |  |  |  |  |  |  |  |  |  |  |  |
| Bachelor’s degree / higher | 33.2%  (42 892) | 41.3%  (38 244) | 26.5%  (36 463) | 35.2%  (64 509) | 19.1%  (205) | 24.7%  (287) | 18.8%  (408) | 25.5%  (327) | 21.0%  (143) | 26.8%  (210) | 13.5%  (376) | 17.4%  (391) |
| Some college / technical vocation | 31.3%  (30 269) | 31.9%  (29 573) | 39.1%  (53 868) | 39.0%  (71 503) | 28.4%  (304) | 31.5%  (366) | 30.4%  (660) | 31.7%  (407) | 26.8%  (182) | 30.8%  (241) | 19.3%  (536) | 21.4%  (480) |
| High school diploma / equivalent | 22.1%  (17 668) | 16.0%  (14 821) | 24.0%  (33 029) | 18.4%  (33 737) | 26.1%  (280) | 21.8%  (253) | 21.7%  (470) | 17.3%  (222) | 27.3%  (185) | 19.8%  (155) | 21.3%  (594) | 17.4%  (390) |
| Less than high school | 12.9%  (410) | 10.2%  (9 460) | 9.9%  (13 558) | 7.0%  (12 789) | 24.9%  (267) | 20.4%  (237) | 28.0%  (608) | 24.5%  (314) | 24.2%  (164) | 21.7%  (170) | 15.3%  (425) | 12.0%  (268) |
| Unstated | 0.7%  (901) | 0.6%  (566) | 0.5%  (705) | 0.5%  (860) | 1.6%  (17) | 1.6%  (18) | 1.1%  (24) | 1.1%  (14) | 0.6%  (5) | 0.9%  (7) | 30.5%  (847) | 31.8%  (714) |
|  |  |  |  |  |  |  |  |  |  |  |  |  |
| **Ethnicity** |  |  |  |  |  |  |  |  |  |  |  |  |
| White | 72.9%  (100 055) | 75.6%  (70 031) | 75.2%  (103 472) | 77.6%  (142 329) | 67.1%  (719) | 69.2%  (803) | 70.6%  (1 530) | 69.4%  (891) | 67.7%  (459) | 67.1%  (525) | 36.8%  (1 022) | 36.9%  (828) |
|  |  |  |  |  |  |  |  |  |  |  |  |  |
| **Income adequacy** | |  |  |  |  |  |  |  |  |  |  |  |
| Very easy / easy | 36.4%  (49 933) | 38.4%  (35 551) | 26.8%  (36 896) | 28.3%  (51 900) | 28.0%  (300) | 28.1%  (326) | 25.2%  (546) | 26.6%  (342) | 18.8%  (127) | 20.1%  (157) | 11.9%  (332) | 11.4%  (255) |
| Neither easy nor difficult | 33.3%  (45 681) | 33.0%  (30 571) | 34.1%  (46 902) | 33.8%  (61 914) | 33.3%  (357) | 31.7%  (368) | 33.2%  (720) | 31.8%  (408) | 32.9%  (223) | 31.2%  (244) | 15.2%  (421) | 15.4%  (346) |
| Very difficult / difficult | 27.4%  (37 571) | 26.1%  (24 148) | 36.0%  (49 552) | 35.2%  (64 572) | 33.8%  (362) | 35.6%  (413) | 38.4%  (834) | 38.4%  (493) | 44.4%  (301) | 45.5%  (356) | 13.3%  (368) | 13.3%  (299) |
| Unstated | 2.9%  (4 035) | 2.5%  (2 394) | 3.1%  (4 271) | 2.7%  (4 738) | 4.9%  (53) | 4.7%  (54) | 3.2%  (71) | 3.2%  (41) | 3.9%  (27) | 3.3%  (26) | 59.7%  (1 656) | 60.0%  (1 343) |
|  |  |  |  |  |  |  |  |  |  |  |  |  |

**Supplemental Table S4: Logistic regression model including covariate output examining past 30-day cannabis use (n=281 533)**

|  |  | **Odds of past 30-day cannabis consumption** | |
| --- | --- | --- | --- |
|  | **% Past 30-day consumers*** | **AOR (95%CI)** | **P level** |
|  |  |  |  |
| **Gender** |  |  |  |
| Cisgender men | 28.1% | Reference | Reference |
| Cisgender women | 22.0% | 0.68 (0.66-0.70) | <.001 |
| Transgender men | 32.7% | 1.12 (0.94-1.34) | .192 |
| Transgender women | 27.0% | 0.88 (0.73-1.05) | .163 |
| Gender-diverse | 28.4% | 0.88 (0.71-1.10) | .258 |
| Unstated | 30.4% | 1.31 (1.13-1.52) | <.001 |
|  |  |  |  |
| **Country** |  |  |  |
| Canada | 24.5% | Reference | Reference |
| United States | 25.5% | 1.06 (1.03-1.09) | <.001 |
|  |  |  |  |
| **Age** |  |  |  |
| 16-25 | 23.4% | 1.48 (1.41-1.56) | <.001 |
| 26-35 | 34.0% | 2.65 (2.54-2.77) | <.001 |
| 36-45 | 28.7% | 2.06 (1.98-2.15) | <.001 |
| 46-55 | 21.5% | 1.34 (1.29-1.40) | <.001 |
| 56-65 | 17.3% | Reference | Reference |
|  |  |  |  |
| **Education level** |  |  |  |
| Bachelor’s degree / higher | 19.8% | Reference | Reference |
| Some college / technical vocation | 27.4% | 1.70 (1.65-1.76) | <.001 |
| High school diploma / equivalent | 29.4% | 1.86 (1.79-1.93) | <.001 |
| Less than high school | 24.0% | 1.42 (1.35-1.51) | <.001 |
| Unstated | 19.9% | 1.05 (0.89-1.24) | .581 |
|  |  |  |  |
| **Race / ethnicity** |  |  |  |
| White | 25.3% | Reference | Reference |
| Other / mixed / unstated | 24.9% | 0.91 (0.88-0.94) | <.001 |
|  |  |  |  |
| **Income adequacy** |  |  |  |
| Very easy / easy | 23.4% | Reference | Reference |
| Neither easy nor difficult | 25.0% | 1.04 (1.01-1.08) | .020 |
| Very difficult / difficult | 27.6% | 1.16 (1.11-1.20) | <.001 |
| Unstated | 21.0% | 0.77 (0.71-0.83) | <.001 |
|  |  |  |  |

*Unadjusted percentage.

**Supplemental Table S5: Logistic regression model including covariate output examining daily cannabis use (n=281 533)**

|  |  | **Odds of daily cannabis consumption** | |
| --- | --- | --- | --- |
|  | **% Daily consumers*** | **AOR (95%CI)** | **P level** |
|  |  |  |  |
| **Gender** |  |  |  |
| Cisgender men | 14.1% | Reference | Reference |
| Cisgender women | 11.7% | 0.74 (0.72-0.77) | <.001 |
| Transgender men | 14.2% | 0.89 (0.72-1.11) | .295 |
| Transgender women | 12.2% | 0.76 (0.59-1.00) | .047 |
| Gender-diverse | 15.4% | 0.96 (0.73-1.26) | .760 |
| Unstated | 20.3% | 1.63 (1.37-1.94) | <.001 |
|  |  |  |  |
| **Country** |  |  |  |
| Canada | 11.9% | Reference | Reference |
| United States | 13.5% | 1.20 (1.16-1.24) | <.001 |
|  |  |  |  |
| **Age** |  |  |  |
| 16-25 | 11.3% | 1.20 (1.12-1.28) | <.001 |
| 26-35 | 18.1% | 2.44 (2.31-2.56) | <.001 |
| 36-45 | 14.9% | 1.93 (1.83-2.04) | <.001 |
| 46-55 | 11.2% | 1.31 (1.24-1.39) | <.001 |
| 56-65 | 8.9% | Reference | Reference |
|  |  |  |  |
| **Education level** |  |  |  |
| Bachelor’s degree / higher | 7.1% | Reference | Reference |
| Some college / technical vocation | 15.0% | 2.44 (2.33-2.56) | <.001 |
| High school diploma / equivalent | 17.7% | 3.01 (2.85-3.17) | <.001 |
| Less than high school | 12.6% | 2.25 (2.08-2.42) | <.001 |
| Unstated | 11.5% | 1.54 (1.23-1.92) | <.001 |
|  |  |  |  |
| **Race / ethnicity** |  |  |  |
| White | 13.0% | Reference | Reference |
| Other / mixed / unstated | 13.0% | 0.95 (0.91-1.00) | .034 |
|  |  |  |  |
| **Income adequacy** |  |  |  |
| Very easy / easy | 10.1% | Reference | Reference |
| Neither easy nor difficult | 13.0% | 1.20 (1.15-1.26) | <.001 |
| Very difficult / difficult | 15.9% | 1.43 (1.37-1.50) | <.001 |
| Unstated | 12.7% | 1.03 (0.93-1.14) | .595 |
|  |  |  |  |

*Unadjusted percentage.

**Supplemental Table S6: Reasons for cannabis use among past 12-month consumers*, by gender identity (n=53 297)****

|  | **Medical reasons** | **Exclusively recreational reasons** |
| --- | --- | --- |
|  |  |  |
|  |  |  |
| Cisgender men | 45.4% (12 544) | 54.6% (15 079) |
| Cisgender women | 49.0% (12 055) | 51.0% (12 531) |
| Transgender men | 52.8% (121) | 47.2% (108) |
| Transgender women | 50.6% (218) | 49.4% (212) |
| Gender-diverse | 64.2% (82) | 35.8% (46) |
| Unstated | 47.8% (146) | 52.2% (159) |
|  |  |  |

*Among half of past 12-month consumers in Waves 3 and 4, and all past 12-month consumers in Waves 5 and 6

**2 900 respondents dropped due to ‘don’t know’ and ‘refused’.

**Supplemental Table S7: Logistic regression model including covariate output examining self-reported perceived addiction to cannabis among past 12-month consumers (n=94 211)***

|  |  | **Odds of very addicted***** | |  | **Odds of a little addicted***** | |  | **Odds of don’t know***** | |
| --- | --- | --- | --- | --- | --- | --- | --- | --- | --- |
|  | **% Very addicted**** | **AOR (95%CI)** | **P level** | **% A little addicted**** | **AOR (95%CI)** | **P level** | **% Don’t know**** | **AOR (95%CI)** | **P level** |
|  |  |  |  |  |  |  |  |  |  |
|  |  |  |  |  |  |  |  |  |  |
| **Gender** |  |  |  |  |  |  |  |  |  |
| Cisgender men | 9.8% | Reference | Reference | 24.0% | Reference | Reference | 3.6% | Reference | Reference |
| Cisgender women | 6.0% | 0.57 (0.53-0.62) | <.001 | 16.4% | 0.61 (0.58-0.64) | <.001 | 3.0% | 0.73 (0.65-0.82) | <.001 |
| Transgender men | 12.4% | 1.32 (0.84-2.08) | .232 | 23.1% | 0.94 (0.69-1.26) | .656 | 5.0% | 1.29 (0.67-2.47) | .445 |
| Transgender women | 12.5% | 1.32 (0.87-1.99) | .190 | 22.2% | 0.86 (0.61-1.21) | .391 | 1.6% | 0.40 (0.19-0.83) | .013 |
| Gender-diverse | 5.5% | 0.47 (0.24-0.91) | .025 | 24.2% | 0.79 (0.56-1.12) | .187 | 2.5% | 0.53 (0.19-1.51) | .237 |
| Unstated | 16.2% | 3.27 (2.17-4.94) | <.001 | 17.3% | 1.43 (0.96-2.11) | .077 | 36.2% | 5.77 (3.86-8.63) | <.001 |
|  |  |  |  |  |  |  |  |  |  |
| **Country** |  |  |  |  |  |  |  |  |  |
| Canada | 7.4% | Reference | Reference | 22.1% | Reference | Reference | 3.5% | Reference | Reference |
| United States | 8.5% | 0.99 (0.91-1.07) | .801 | 19.6% | 0.77 (0.73-0.82) | <.001 | 3.7% | 1.05 (0.94-1.18) | .370 |
|  |  |  |  |  |  |  |  |  |  |
| **Age** |  |  |  |  |  |  |  |  |  |
| 16-25 | 7.9% | 3.14 (2.63-3.74) | <.001 | 23.2% | 2.60 (2.36-2.86) | <.001 | 4.3% | 1.86 (1.50-2.31) | <.001 |
| 26-35 | 10.2% | 3.41 (2.91-4.00) | <.001 | 23.2% | 2.10 (1.92-2.29) | <.001 | 4.2% | 2.15 (1.78-2.59) | <.001 |
| 36-45 | 9.8% | 3.35 (2.86-3.93) | <.001 | 22.1% | 2.00 (1.83-2.18) | <.001 | 3.9% | 2.04 (1.69-2.47) | <.001 |
| 46-55 | 6.7% | 2.16 (1.81-2.57) | <.001 | 16.5% | 1.35 (1.23-1.49) | <.001 | 2.7% | 1.33 (1.08-1.64) | .008 |
| 56-65 | 3.4% | Reference | Reference | 13.6% | Reference | Reference | 2.2% | Reference | Reference |
|  |  |  |  |  |  |  |  |  |  |
| **Education level** |  |  |  |  |  |  |  |  |  |
| Bachelor’s degree / higher | 9.1% | Reference | Reference | 20.5% | Reference | Reference | 2.5% | Reference | Reference |
| Some college / technical vocation | 6.7% | 0.60 (0.54-0.66) | <.001 | 19.3% | 0.76 (0.71-0.81) | <.001 | 3.0% | 0.93 (0.80-1.08) | .355 |
| High school diploma / equivalent | 8.9% | 0.73 (0.65-0.81) | <.001 | 21.6% | 0.77 (0.71-0.83) | <.001 | 4.2% | 1.18 (1.00-1.39) | .046 |
| Less than high school | 9.1% | 0.87 (0.75-1.01) | .070 | 21.8% | 0.82 (0.74-0.91) | <.001 | 5.7% | 1.67 (1.34-2.08) | <.001 |
| Unstated | 8.2% | 1.02 (0.57-1.81) | .950 | 19.7% | 1.31 (0.80-2.14) | .284 | 31.0% | 3.14 (1.95-5.06) | <.001 |
|  |  |  |  |  |  |  |  |  |  |
| **Race / ethnicity** |  |  |  |  |  |  |  |  |  |
| White | 7.3% | Reference | Reference | 19.5% | Reference | Reference | 3.2% | Reference | Reference |
| Other / mixed / unstated | 10.7% | 1.51 (1.37-1.65) | <.001 | 23.3% | 1.21 (1.13-1.29) | <.001 | 4.8% | 1.31 (1.15-1.48) | <.001 |
|  |  |  |  |  |  |  |  |  |  |
| **Income adequacy** |  |  |  |  |  |  |  |  |  |
| Very easy / easy | 20.6% | Reference | Reference | 10.2% | Reference | Reference | 2.2% | Reference | Reference |
| Neither easy nor difficult | 21.0% | 0.58 (0.52-0.65) | <.001 | 6.4% | 0.58 (0.52-0.65) | .410 | 3.7% | 1.51 (1.29-1.76) | <.001 |
| Very difficult / difficult | 20.3% | 0.77 (0.69-0.85) | <.001 | 8.2% | 0.98 (0.91-1.05) | .548 | 3.3% | 1.37 (1.16-1.61) | <.001 |
| Unstated | 13.8% | 0.47 (0.35-0.63) | <.001 | 6.5% | 0.61 (0.49-0.74) | <.001 | 23.8% | 5.98 (4.73-7.57) | <.001 |
|  |  |  |  |  |  |  |  |  |  |
| **Cannabis use** |  |  |  |  |  |  |  |  |  |
| Yearly consumer | 1.8% | Reference | Reference | 5.0% | Reference | Reference | 1.9% | Reference | Reference |
| Monthly / weekly consumer | 5.8% | 3.97 (3.35-4.70) | <.001 | 20.3% | 5.14 (4.65-5.68) | <.001 | 3.2% | 2.28 (1.94-2.69) | <.001 |
| Daily / almost daily consumer | 14.7% | 17.33 (14.78-20.33) | <.001 | 31.4% | 13.04 (11.81-14.41) | <.001 | 5.2% | 5.06 (4.31-5.93) | <.001 |
|  |  |  |  |  |  |  |  |  |  |

*647 dropped due to refused.

**Unadjusted percentage.

***‘Not at all addicted’ is the reference group.

**Supplemental Table S8: Personal effects of cannabis use reported by past 12-month consumers, by gender identity**

|  | **Quality of life** | | **Mental health** | | **Physical health** | | **Friendships and**  **social life** | | **Family life** | | **Studies** | | **Work** | |
| --- | --- | --- | --- | --- | --- | --- | --- | --- | --- | --- | --- | --- | --- | --- |
|  | n = 89 672 | | n = 89 581 | | n = 88 820 | | n = 86 982 | | n = 86 708 | | n = 75 383 | | n = 79 422 | |
|  | **Positive** | **Negative** | **Positive** | **Negative** | **Positive** | **Negative** | **Positive** | **Negative** | **Positive** | **Negative** | **Positive** | **Negative** | **Positive** | **Negative** |
|  |  |  |  |  |  |  |  |  |  |  |  |  |  |  |
| Cisgender men | 48.8%  (22 682) | 4.6%  (2 124) | 46.2%  (21 448) | 5.4%  (2 523) | 31.7%  (14 649) | 6.5%  (2 984) | 29.6%  (13 407) | 5.1%  (2 309) | 22.7%  (10 228) | 6.3%  (2 854) | 21.5%  (8 663) | 6.6%  (2 651) | 21.1%  (8 878) | 6.3%  (2 654) |
| Cisgender women | 48.8%  (20 008) | 3.4%  (1 403) | 49.5%  (20 273) | 4.6%  (1 870) | 34.1%  (13 836) | 5.5%  (2 226) | 21.7%  (8 584) | 3.4%  (1 344) | 20.5%  (8 153) | 4.5%  (1 783) | 14.1%  (4 703) | 5.4%  (1 782) | 14.5%  (5 131) | 4.1%  (1 456) |
| Transgender men | 53.2%  (219) | 6.2%  (26) | 57.0%  (236) | 8.9%  (37) | 32.0%  (127) | 8.9%  (36) | 32.5%  (128) | 7.8%  (31) | 24.5%  (99) | 11.0%  (45) | 22.4%  (80) | 8.4%  (31) | 21.1%  (77) | 8.0%  (29) |
| Transgender women | 48.9%  (352) | 7.8%  (56) | 52.2%  (379) | 9.6%  (70) | 33.5%  (236) | 9.4%  (66) | 26.2%  (182) | 7.5%  (52) | 29.3%  (203) | 12.0%  (84) | 27.7%  (178) | 6.6%  (43) | 26.2%  (169) | 7.8%  (51) |
| Gender-diverse | 62.7%  (162) | 2.1%  (6) | 64.2%  (166) | 4.4%  (12) | 44.7%  (113) | 5.5%  (14) | 30.4%  (76) | 1.9%  (5) | 18.8%  (47) | 6.1%  (16) | 19.0%  (41) | 6.0%  (13) | 18.7%  (43) | 2.9%  (7) |
| Unstated | 35.1%  (262) | 4.6%  (35) | 36.2%  (260) | 5.8%  (42) | 28.3%  (195) | 7.0%  (49) | 27.0%  (196) | 6.7%  (49) | 26.4%  (189) | 7.6%  (55) | 20.6%  (145) | 5.1%  (37) | 23.1%  (161) | 5.4%  (38) |
|  |  |  |  |  |  |  |  |  |  |  |  |  |  |  |

*8 665 dropped from friendships and social life; 6 783 dropped from physical health; 6 196 dropped from mental health; 8 939 dropped from family life; 16 942 dropped from work; 21 782 dropped from studies; and 4 759 dropped from quality of life due to ‘refused’ or ‘not applicable’.

**Supplemental Table S9: Linear regression model including covariate output examining personal impacts of cannabis use among past 12-month consumers (n=91 231)***

|  | **Mean (± Std Error)**** | **β (95%CI)** | **P level** |
| --- | --- | --- | --- |
|  |  |  |  |
| **Gender** |  |  |  |
| Cisgender men | 8.16 (± 0.02) | Reference | Reference |
| Cisgender women | 8.06 (± 0.02) | -0.01 ([-0.07]-0.04) | .671 |
| Transgender men | 8.15 (± 0.20) | -0.02 ([-0.39]-0.35) | .916 |
| Transgender women | 8.21 (± 0.19) | 0.11 ([-0.25]-0.46) | .563 |
| Gender-diverse | 8.63 (± 0.17) | 0.53 (0.21-0.86) | .001 |
| Unstated | 7.30 (± 0.20) | -0.50 ([-0.91]-[-0.10]) | .014 |
|  |  |  |  |
| **Country** |  |  |  |
| Canada | 7.67 (± 0.02) | Reference | Reference |
| United States | 8.33 (± 0.02) | 0.57 (0.52-0.62) | <.001 |
|  |  |  |  |
| **Age** |  |  | <.001 |
| 16-25 | 7.90 (± 0.04) | 0.44 (0.34-0.53) | <.001 |
| 26-35 | 8.40 (± 0.03) | 0.72 (0.64-0.80) | <.001 |
| 36-45 | 8.36 (± 0.03) | 0.69 (0.61-0.76) | <.001 |
| 46-55 | 7.96 (± 0.03) | 0.33 (0.24-0.41) | <.001 |
| 56-65 | 7.63 (± 0.03) | Reference | Reference |
|  |  |  |  |
| **Education level** |  |  |  |
| Bachelor’s degree / higher | 8.21 (± 0.03) | Reference | Reference |
| Some college / technical vocation | 8.20 (± 0.02) | -0.05 ([-0.12]-0.01) | .124 |
| High school diploma / equivalent | 8.08 (± 0.03) | -0.20 ([-0.28]-[-0.12]) | <.001 |
| Less than high school | 7.72 (± 0.05) | -0.41 ([-0.52]-[-0.30]) | <.001 |
| Unstated | 7.02 (± 0.18) | -0.79 ([-1.18]-[-0.40]) | <.001 |
|  |  |  |  |
| **Race / ethnicity** |  |  |  |
| White | 8.10 (± 0.02) | Reference | Reference |
| Other / mixed / unstated | 8.13 (± 0.03) | -0.03 ([-0.10]-0.04) | .381 |
|  |  |  |  |
| **Income adequacy** |  |  |  |
| Very easy / easy | 8.23 (± 0.03) | Reference | Reference |
| Neither easy nor difficult | 8.08 (± 0.02) | -0.15 ([-0.22]-[-0.07]) | <.001 |
| Very difficult / difficult | 8.09 (± 0.02) | -0.16 ([-0.24]-[-0.09]) | <.001 |
| Unstated | 7.48 (± 0.86) | -0.61 ([-0.78]-[-0.44]) | <.001 |
|  |  |  |  |
| **Cannabis use** |  |  |  |
| Yearly consumer | 7.30 (± 0.02) | Reference | Reference |
| Monthly / weekly consumer | 8.10 (± 0.02) | 0.74 (0.68-0.81) | <.001 |
| Daily / almost daily consumer | 8.66 (±0.03) | 1.32 (1.26-1.39) | <.001 |
|  |  |  |  |

*Respondents dropped due to ‘refused’ or ‘not applicable’ for all items included in the index.

**Unadjusted mean comes from the index from 0-14 of all seven of the personal effect questions.

**Supplemental Table S10: Comfort consuming cannabis in social settings among all respondents, by gender identity**

|  | **Around friends** | **Around partner** | **Around parents** | **Around co-workers** | **In public** | **Around children** |
| --- | --- | --- | --- | --- | --- | --- |
|  | n = 252 590 | n = 238 827 | n = 235 648 | n = 229 376 | n = 251 571 | n = 242 927 |
|  |  |  |  |  |  |  |
|  |  |  |  |  |  |  |
| Cisgender men | 52.7% (65 708) | 48.4% (57 139) | 29.0% (33 831) | 27.1% (31 256) | 24.7% (30 784) | 14.1% (16 923) |
| Cisgender women | 50.0% (61 105) | 51.7% (59 573) | 24.6% (27 924) | 16.9% (18 349) | 14.8% (18 031) | 7.5% (8 777) |
| Transgender men | 56.8% (563) | 54.1% (501) | 30.9% (296) | 24.0% (222) | 24.7% (246) | 14.6% (138) |
| Transgender women | 55.4% (1 087) | 50.4% (932) | 30.4% (580) | 24.7% (439) | 24.1% (472) | 15.1% (284) |
| Gender-diverse | 61.7% (377) | 61.8% (340) | 29.9% (179) | 18.3% (96) | 18.9% (116) | 8.5% (49) |
| Unstated | 26.0% (544) | 24.0% (506) | 18.5% (391) | 17.4% (359) | 16.1% (338) | 12.3% (253) |
|  |  |  |  |  |  |  |

*49 989 dropped from around parents; 39 841 dropped from around partner; 28 640 dropped from around friends; 53 769 dropped from around co-workers; 37 876 dropped from around children; and 29 917 dropped from in public due to ‘refused’ or ‘not applicable’.

**Supplemental Table S11: Linear regression model including covariate output examining social norms via comfort consuming cannabis, among all respondents (n=258 428)***

|  | **Mean (± Std Error)**** | **β (95%CI)** | **P level** |
| --- | --- | --- | --- |
|  |  |  |  |
| **Gender** |  |  |  |
| Cisgender men | 1.85 (± 0.01) | Reference | Reference |
| Cisgender women | 1.55 (± 0.01) | -0.21 ([-0.23]-[-0.19]) | <.001 |
| Transgender men | 1.94 (± 0.08) | 0.03 ([-0.11]-0.17) | .668 |
| Transgender women | 1.88 (± 0.07) | 0.07 ([-0.06]-0.19) | .282 |
| Gender-diverse | 1.84 (± 0.08) | -0.05 ([-0.18]-0.09) | .518 |
| Unstated | 1.06 (± 0.06) | -0.43 ([-0.54]-[-0.32]) | <.001 |
|  |  |  |  |
| **Country** |  |  |  |
| Canada | 1.55 (± 0.01) | Reference | Reference |
| United States | 1.77 (± 0.01) | 0.20 (0.18-0.22) | <.001 |
|  |  |  |  |
| **Age** |  |  |  |
| 16-25 | 1.51 (± 0.01) | 0.19 (0.16-0.22) | <.001 |
| 26-35 | 2.15 (± 0.01) | 0.60 (0.57-0.63) | <.001 |
| 36-45 | 1.95 (± 0.01) | 0.49 (0.47-0.52) | <.001 |
| 46-55 | 1.54 (± 0.01) | 0.21 (0.18-0.23) | <.001 |
| 56-65 | 1.26 (± 0.01) | Reference | Reference |
|  |  |  |  |
| **Education level** |  |  |  |
| Bachelor’s degree / higher | 1.59 (± 0.01) | Reference | Reference |
| Some college / technical vocation | 1.76 (± 0.01) | 0.11 (0.08-0.13) | <.001 |
| High school diploma / equivalent | 1.86 (± 0.01) | 0.19 (0.16-0.22) | <.001 |
| Less than high school | 1.50 (± 0.02) | -0.02 ([-0.06]-0.02]) | .319 |
| Unstated | 0.83 (± 0.06) | -0.25 ([-0.36]-[-0.14]) | <.001 |
|  |  |  |  |
| **Race / ethnicity** |  |  |  |
| White | 1.72 (± 0.01) | Reference | Reference |
| Other / mixed / unstated | 1.62 (± 0.01) | -0.08 ([-0.10]-[-0.06]) | <.001 |
|  |  |  |  |
| **Income adequacy** |  |  |  |
| Very easy / easy | 1.80 (± 0.01) | Reference | Reference |
| Neither easy nor difficult | 1.58 (± 0.01) | -0.27 ([-0.29]-[-0.24]) | <.001 |
| Very difficult / difficult | 1.79 (± 0.01) | -0.14 ([-0.16]-[-0.11]) | <.001 |
| Unstated | 0.99 (± 0.03) | -0.66 ([-0.72]-[0.61]) | <.001 |
|  |  |  |  |
| **Cannabis use** |  |  |  |
| Yearly consumer | 2.11 (± 0.01) | Reference | Reference |
| Monthly / weekly consumer | 2.68 (± 0.01) | 0.51 (0.47-0.55) | <.001 |
| Daily / almost daily consumer | 3.23 (± 0.01) | 1.04 (0.99-1.08) | <.001 |
| Non-consumer | 1.09 (± 0.01) | -0.99 ([-1.02]-[-0.96]) | <.001 |
|  |  |  |  |

*Respondents dropped due to ‘refused’ or ‘not applicable’ for all items included in the index.

**Unadjusted mean comes from the index from 0-6 of all six of the comfort consuming cannabis question.
